# Using Gene Expression Analysis to Understand Complex Autoimmune Skin Disease Patients: A Series of Four Canine Cutaneous Lupus Erythematosus Cases

**DOI:** 10.3389/fvets.2022.778934

**Published:** 2022-02-24

**Authors:** Alice A. Amudzi, Cesar Piedra-Mora, Diana Junyue Ma, Neil B. Wong, Clement N. David, Nicholas A. Robinson, Ramón M. Almela, Jillian M. Richmond

**Affiliations:** ^1^Dermatology Department, University of Massachusetts Chan Medical School, Worcester, MA, United States; ^2^Pathology Department, Tufts Cummings School of Veterinary Medicine, North Grafton, MA, United States; ^3^NanoString Technologies, Inc., Seattle, WA, United States; ^4^Department of Clinical Sciences, Tufts Cummings School of Veterinary Medicine, North Grafton, MA, United States

**Keywords:** systemic lupus erythematosus, immunopathogenesis, interface dermatitis, cytokine, chemokine, comparative immunology, canine (dog), cutaneous lupus erythematosus (CLE)

## Abstract

Cutaneous Lupus Erythematosus (CLE) is an autoimmune skin disease that occurs in almost two-thirds of people with Systemic Lupus Erythematosus (SLE) and can exist as its own entity. Despite its negative impact on the quality of life of patients, lupus pathogenesis is not fully understood. In recent years, the role of gene expression analysis has become important in understanding cellular functions and disease causation within and across species. Interestingly, dogs also develop CLE, providing a spontaneous animal model of disease. Here, we present a targeted transcriptomic analysis of skin biopsies from a case series of four dogs with complex autoimmunity with suspected CLE. We identified 92 differentially expressed genes (DEGs), including type 1 interferon, B cell, and T cell-related genes, in the four cases compared to healthy skin margin controls. Additionally, we compared our results with existing CLE datasets from humans and mice and found that humans and canines share 49 DEGs, whereas humans and mice shared only 25 DEGs in our gene set. Immunohistochemistry of IFNG and CXCL10, two of the most highly upregulated inflammatory mediators, confirmed protein-level expression and revealed immune cells as the primary source of CXCL10 in dogs with SLE, whereas keratinocytes stained strongly for CXCL10 in dogs without SLE. We propose that gene expression analysis may aid the diagnosis of complex autoimmune skin diseases and that dogs may provide important insights into CLE and SLE pathogeneses, or more broadly, skin manifestations during systemic autoimmunity.

## Introduction

Cutaneous Lupus Erythematosus (CLE) is a complex autoimmune disease that primarily involves the skin (1, 2). Like systemic lupus erythematosus (SLE), CLE occurs predominantly in women between 20 and 40 years (3). The worldwide incidence of CLE is estimated at 4.2/100,000 persons (4). African-Americans have 3–5 fold increased susceptibility of developing CLE (5). When left untreated, CLE can result in permanent scarring alopecia, dyspigmentation, gross facial deformity, macular hyperpigmentation, and rarely squamous cell carcinoma (6–8), having a significant negative impact on the quality of life of patients (9).

While there is a close relationship between CLE and SLE, the connection between skin inflammation and systemic disease is unclear. Some proposed factors influencing CLE pathogenesis include familial predisposition with strong associations with HLA-DRB1^*^16, HLA-DR2, HLA-DR3, HLA-B7, and HLA-B8 (10), environmental triggers (UV light, silica dust, infections), and lifestyle (smoking) (11). Some studies suggest that when one or more of these factors are triggered in a susceptible individual, immune pathways are activated resulting in tissue damage and perpetuation of the inflammatory cycle. This also leads to chronic TGFβ signaling, which promotes scarring (12).

**Figure 1:**
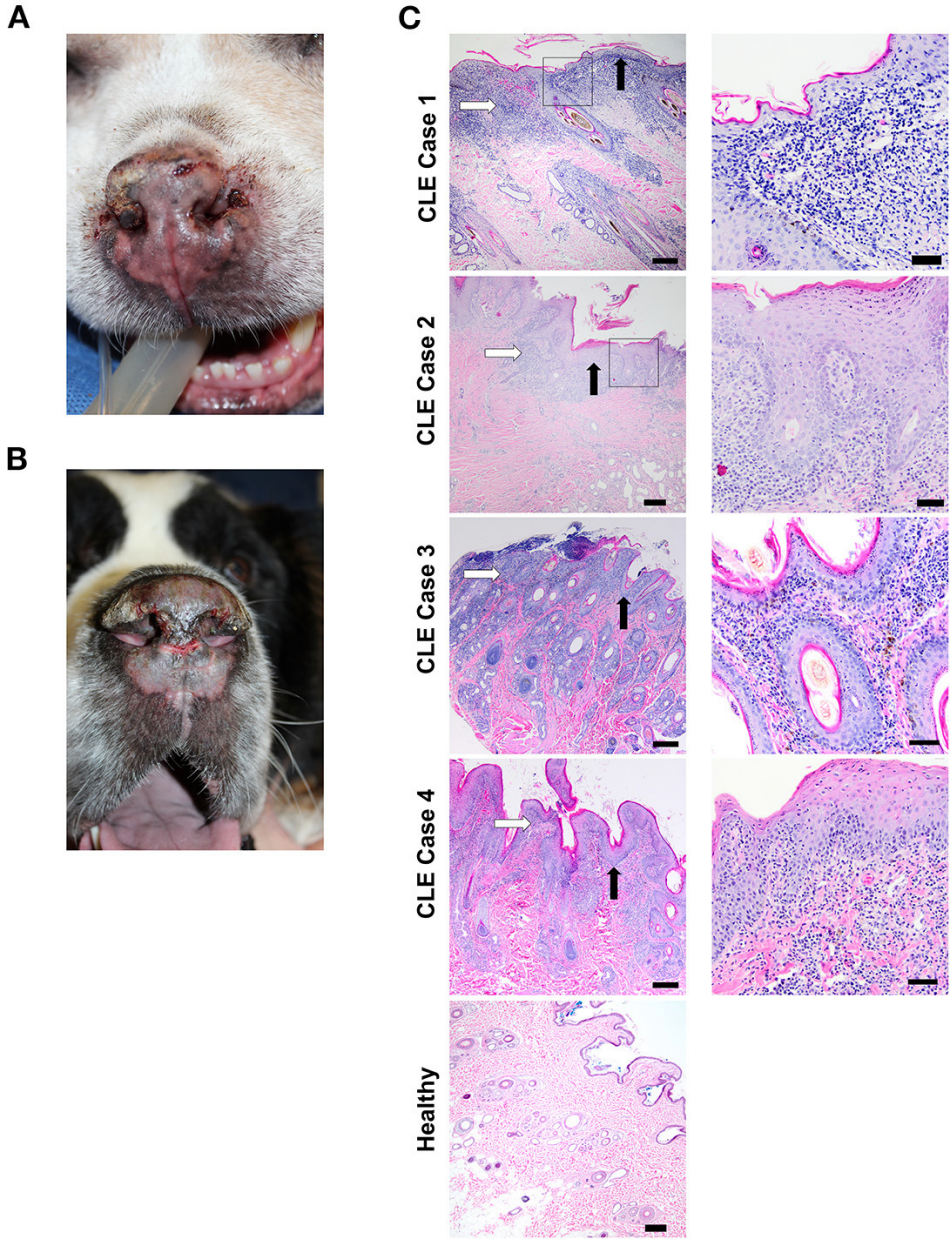
Clinical features and histopathological findings of canine CLE cases. **(A)** Nasal planum exhibits scarring, dyspigmentation, loss of nasal architecture (loss of the normal cobblestone appearance), erosions/ulcerations, and hemorrhagic crusts. **(B)** Nasal planum exhibits atrophy and loss of nasal architecture, symmetrical erosions/ulcerations, and scarring on the dorsal muzzle. The dermatopathological hallmarks of canine CLE include a lymphocytic-rich interface dermatitis with basal keratinocyte damage that obscures the dermal-epidermal junction (DEJ). **(C)** CLE case 1, 2, 3, 4, and healthy skin margin H&E photomicrographs (left, 4x objective, right 20X objective). Basement membrane thickening, obscuring of the DEJ with basal keratinocyte damage (black arrows), and lymphocytic infiltration (white arrows) are observed in all cases. [Scale bars 4x objective images = 200 μM, 20x objective images = 50 μm].

Interestingly, CLE and SLE can also occur spontaneously in dogs and have clinical and histologic features similar to human disease (13). Comparative studies of dogs, humans, and mice have revealed similarities in inflammatory gene signatures in other disease processes like cancer, and clinical trials are more efficiently run in dogs due to their condensed lifespan (14, 15). Interface dermatitis involving the dermal-epidermal junction also occurs in all three species in autoimmune-mediated diseases, including CLE (16). Further, it is evident that genetics plays a role in canine CLE, as certain breeds are more predisposed to developing the disease than others. Examples of clinical features of canine CLE, including erythema, scaling, erosions, ulcerations, and crusting involving the nasal planum, pinna, periorbital skin, and muzzle with scarring, are presented in Figures 1A,B.

The “One Health Approach,” which is a collaborative, multisectoral, and transdisciplinary school of thought, recognizes the interconnection between the health of people and animals and their shared environment and emphasizes the need to combine investigative methods and animal models for studying human diseases (17). We (Garelli et al.) recently performed a comparative transcriptomics study of canine and human discoid lupus erythematosus (DLE) and found conserved gene expression patterns (18). The objective of this study was to determine if we can diagnose unclear CLE cases using gene expression profiling. Here, we present four cases of canine CLE that had an unclear skin diagnosis at the time of biopsy, including two with concomitant systemic autoimmunity suspected to be SLE. Using NanoString technology and comparative analyses of previously published human and canine datasets, we found that these cases were most likely chronic CLE (CCLE), with a 62.03% overlap with human CCLE. Our data support a conserved CLE immune gene expression set in dogs and humans and identify potential predictors of systemic involvement.

## Case Presentations

### Case 1

A 7-year-old male neutered German Shepherd Dog presented with a 1-year history of progressive superficial crusting lesions along the skin of the dorsal thoracolumbar spine with a focal 1 cm area of alopecia. A complete blood count showed thrombocytopenia, for which the dog received azathioprine after responding poorly to prednisone. In addition, the dog received broad-spectrum antibiotics for staphylococcal infection, and a skin biopsy revealed multifocal lymphohistiocytic interface dermatitis (**white arrow**) and folliculitis with a moderate lichenoid band of lymphocytes, plasma cells, vacuolization of the basal keratinocytes, and macrophages at the dermal-epidermal and dermal-follicular junctions with frequent loss of the basal cell layer (Figure 1C, CLE case 1). An initial histopathological diagnosis was consistent with chronic CLE.

### Case 2

An 18-year-old male neutered mixed-breed dog with a past medical history of pemphigus foliaceus was presented with thick crusting on the muzzle, periocular region, and inner pinnae, with systemic signs (polyarthritis, fever, lymphadenopathy, anorexia, and lethargy) for which the dog received broad-spectrum antibiotics in addition to azathioprine and prednisone. The dog later developed ulcerative lesions on the nose and footpads. Histopathology of lesional skin revealed lichenoid interface dermatitis (**white arrow**) composed of lymphocytes, plasma cells, histiocytes, and smaller numbers of scattered neutrophils, primarily in the superficial dermis, with rare disorganized apoptotic basal keratinocytes, dermal fibrosis, and hyperkeratosis (black arrow, Figure 1C, CLE case 2). The dog subsequently presented with progressive weight loss and increased liver enzymes and elevated blood urea nitrogen levels that led to the euthanasia of the dog. Initial histopathological diagnosis of lupus vs. pemphigus foliaceus (possibly pemphigus erythematosus).

### Case 3

A 5-year-old male neutered, miniature pinscher dog was presented with early Addison-like crises, pyoderma, and recurrent ulcerative and crusted skin lesions involving the mucocutaneous junctions and legs. The initial histopathological diagnosis was pemphigus foliaceus vs. mucocutaneous pyoderma. The dog received antibiotic treatment (8 weeks cefpodoxime), but the lesions did not fully clear. Two months after, the dog developed additional lesions with some scaling and crusty lesions on pressure points (elbows, hocks, and pinna margins) and central depigmentation on paw pads that clinician based on clinical presentation, which suggested an ischemic vasculopathy that was treated with pentoxifylline. The dog improved, but the lesions did not completely resolve. Lesions persisted for 3 years, and the dog received several courses of antibiotics and prednisone. Repeat biopsy histopathology revealed marked lymphoplasmacytic and histiocytic interface dermatitis of the dermal-epidermal junction and superficial dermis with extension into the mid-dermis at the level of the adnexa, follicular epithelium (multifocal follicular keratosis), and basal cells (Figure 1C, CLE case 3). In addition, there was marked superficial epidermal ulceration with suppurative serocellular crust formation and intralesional cocci and moderate pigmentary incontinence. Re-review of clinical findings by dermatologist included mucocutaneous pyoderma vs. Discoid Lupus Erythematosus (DLE) as possible differential diagnoses, suggesting there was underlying ischemic vasculopathy or CLE.

### Case 4

A 6-year-old female spayed West Highland White Terrier presented severe mucopurulent discharge in both eyes, with heavy crusting and periocular matting of hair. Histology revealed multifocal lymphoplasmacytic and histiocytic lichenoid interface dermatitis with focal ulceration as well as suppurative dermatitis (Figure 1C, CLE case 4). Corneal fibrosis was observed in both eyes in addition to neovascularization, most severe in the right eye. The history above and microscopic findings were suggestive of an immune-mediated process. The dog had a history of hypoadrenocorticism, and an initial histopathological diagnosis suggested DLE vs. mucocutaneous pyoderma vs. SLE.

Additional veterinary care information is provided in Supplementary Document 1.

## Materials and Methods

### Clinical Samples

This retrospective animal study was reviewed and approved by Cummings School of Veterinary Medicine at Tufts University. Diagnostic biopsies were taken at the time of presentation to Tufts Veterinary Clinic and were formalin fixed paraffin embedded (FFPE). After sections were obtained for histopathological diagnoses, tissue blocks were preserved in the biorepository per IACUC approval. For these studies, additional curls and cuts were obtained from the remaining tissue as described in the “Gene Expression Analysis and Case Clustering” methods section.

Our inclusion criteria for archival study samples included interface dermatitis on H&E and clinical features consistent with CLE. Marginal skin tissue from osteosarcoma leg amputations of an 8-year-old female spayed Labrador Retriever, an 11-year-old female spayed Siberian Husky cross, an 11-year-old, castrated male Golden Retriever, a 12-year-old, castrated German Shepherd Dog cross, and a 6-year-old, female spayed Alaskan Malamute, were used as controls (skin tissue had no evidence of disease). Histology slides and pathology reports from all cases were re-examined by a board-certified veterinary pathologist and dermatologist.

### RNA Isolation

RNA was isolated from 30 μm tissue curls using the Qiagen formalin-fixed paraffin-embedded (FFPE) tissue RNeasy kit per the manufacturer's directions and as previously described (18–20). RNA was quantified using a NanoDrop spectrophotometer.

### Gene Expression Analysis and Case Clustering

A customized canine gene list of 160 genes, including skin vs. immune cell-specific cytokines, chemokines, and immune genes, was curated (Supplementary Table 1), and a probe panel was created by staff scientists at NanoString. RNA hybridization was achieved using a BioRad C1000 touch machine. Specimens were loaded into NanoString cartridges and analyzed with a Sprint nCounter. nSolver software was used for all normalization and fold change calculations of canine samples. We used *B2m, Rpl13a, cg14980*, and *hprt* as housekeeping genes. The quality of the RNA was evaluated using the QC parameter in nSolver software, with settings to flag lanes when 0.5 fM positive control is ≤2 SD above the mean of negative controls; none of the lanes returned a QC flag. Advanced analysis for the “Cell Type Score,” which is a summary statistic of the expression of the marker genes for each cell type, was used (21). Any counts under log2 of 5 (y-axis) on the cell type scores are considered undetected. A cell type score < 5 is not detected. Raw data are deposited on Gene Expression Omnibus (GEO) Database under accession # GSE180276.

### H&E and Immunohistochemistry

5 μm sections were used for H&E (Sakura Tissue-Tek DRS autostainer) or IHC using rabbit-anti-canine CXCL10 (US Biological Cat #140923, RRID: AB_2861250), anti-canine IFNγ (US Biological Cat# 363576, RRID: AB_2861251), or isotype control (Biolegend Cat # 910801, RRID: AB_2722735) at 1:50 or 1:100 dilution, respectively (Dako EnVision+ Dual Link System-HRP). Images were taken using an Olympus BX51 microscope with Nikon NIS Elements software version 3.10.

### Canine, Human and Mouse Dataset Alignment and Analyses

The 160 custom canine gene dataset presented here (GSE180276), 755 genes murine cancer immune dataset by Mande et al. (22), and GSE95474 dataset (23) were compared. Common denominator genes were identified in Excel using the formula “=IF(ISERROR(VLOOKUP(A2,$B$2:$B$1001,1,FALSE)),FALSE, TRUE).” Alternate gene names for values that were retrieved as FALSE were queried using GeneCards.org, and naming conventions were matched across all species, which resulted in 160 overlapping genes for canine vs. human (entire canine codeset), and 92 genes overlapping all three species [see also (18)].

### Statistics and Data Visualization

Statistical analyses for the volcano, violin, and box plots were performed in GraphPad Prism version 9.1.2 (24). Shapiro-Wilk normality tests were performed, and normally distributed data were analyzed with a two-tailed student's *t*-test, and non-normally distributed data were analyzed with a two-tailed Mann-Whitney *U*-test to compare healthy to CLE lesional skin. *P*-values < 0.05 were considered significant, with *P* < 0.01 as highly significant. Heatmap of “Cell Type Score” was created with Morpheus software (25). PCA plots and hierarchical clusters were created with ClustVis software (26), BioVenn diagrams were used to illustrate comparisons between species (27) and *p*-values calculated with nSolver (28).

## Results

### Histology of Lesional Skin Reveals Interface Dermatitis and Basement Membrane Thickening

H & E-stained sections from all four cases and healthy controls were used for histopathological analysis (Figure 1C). Cases exhibited marked interface dermatitis, with a predominance of lymphocytic infiltration (**white arrow**) in the skin, thickening of the basement membrane (**black arrow**), vacuolization and apoptosis of basal cells, melanin pigment incontinence, and superficial dermal fibrosis. These features are consistent with CLE. Specimens from the dogs with control skin were all normal.

### Gene Expression Analysis Reveals Substantial Overlap Between Complex Canine CLE and Canine DLE

We isolated RNA from FFPE skin curls from the four (4) canine cases and five (5) healthy margins. We chose NanoString technology to analyze RNA from these FFPE samples because the technology works well for fragmented RNA and avoids amplification, which could introduce a data acquisition bias (29). To ascertain whether the cases presented here aligned with cases with confirmed diagnoses, we performed hierarchical clustering using ClustVis with GSE160260 [canine DLE; dataset originally published in Garelli et al. (18)] and GSE171079 [canine pemphigus including pemphigus erythematosus; dataset originally published in Raef et al. (20)]. Hierarchical clustering of gene expression for the whole panel differentiates between healthy controls and all cases, with a significant overlap between DLE and the cases presented here (Supplementary Figures 1A,B). Principal component analysis (PCA) revealed that all CLE and healthy cases fell within the 95% confidence interval for predicting disease status.

### Key Inflammatory Genes Are Upregulated in CLE Cases vs. Healthy Controls

**Figure 2:**
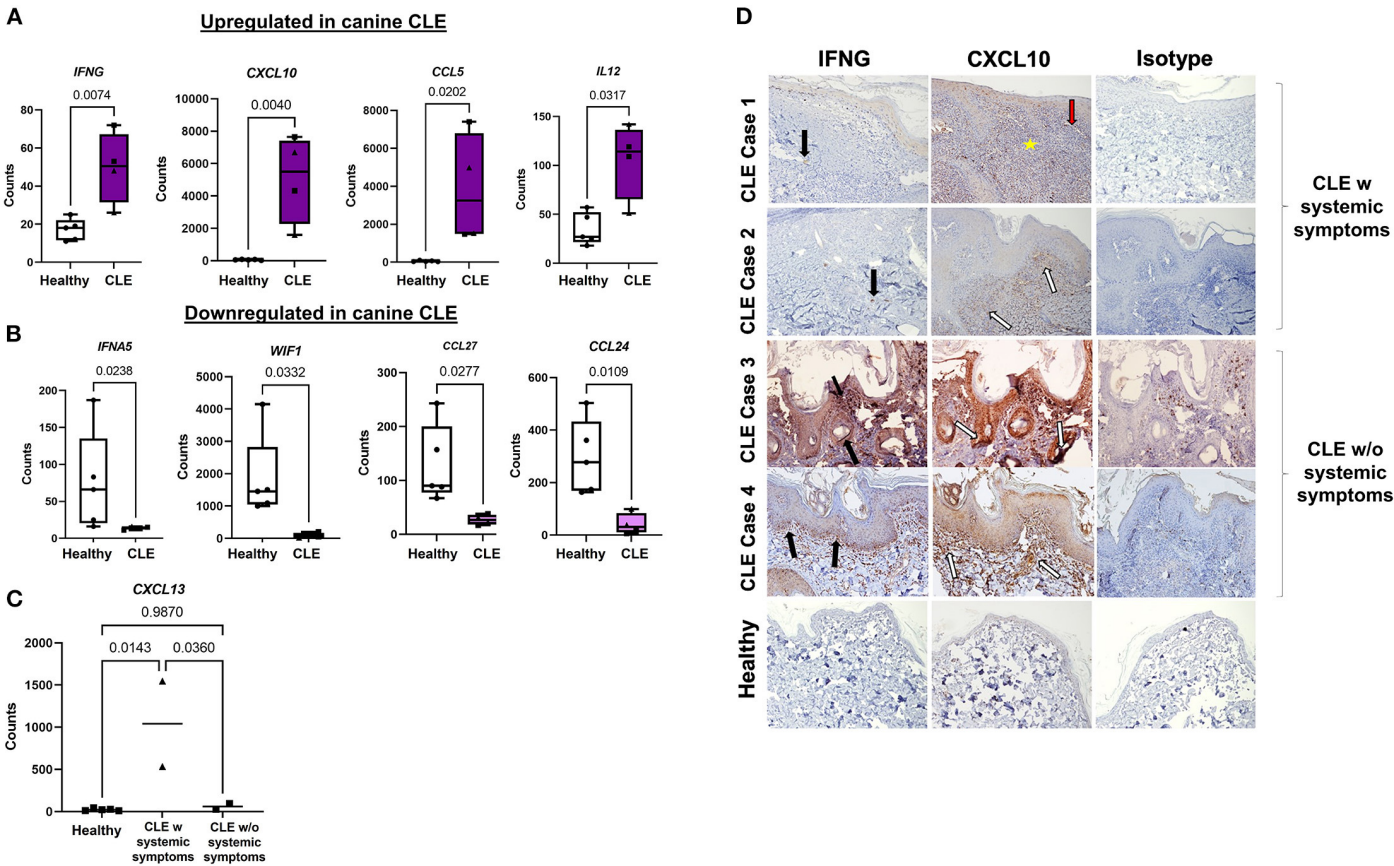
Gene signatures and immunohistochemical features of complex CLE cases. Box plots of **(A)** significantly upregulated and **(B)** downregulated genes in cases vs. controls. **(C)** Examination of *CXCL13* expression in CLE with or without systemic symptoms. **(D)** Immunohistochemistry demonstrates the origin of IFNγ and CXCL10 expression in CLE skin vs. control skin and isotype control. IFNγ (black arrows) and CXCL10 (white arrows) secretion in CLE+systemic symptoms are derived from the immune infiltrate in CLE case 1 and 2, in contrast to keratinocytes at the basal epidermis in CLE case 3 and 4 (10x objective). Perivascular (red arrow-blood vessel) lymphocyte infiltration (yellow star) (w, with, triangle symbols; w/o, without, square symbols).

Ninety-two out of 160 genes were differentially expressed using a significance cutoff of *p* < 0.05, accounting for more than half of the total genes studied (Supplementary Table 2). Seventy genes were highly expressed at a significance cutoff of *p* < 0.01, including *IFNG* (FC = 3.53) and the interferon-related chemokine *CXCL10* (FC = 7.07) (30, 31). Box plots illustrate key upregulated and downregulated genes in healthy and CCLE cases (Figures 2A,B). Thirty-nine out of 92 genes were upregulated using a log FC of >2 at a significance of *p* < 0.05, mainly consisting of inflammatory chemokines *(CXCL10, CCL5, CCL28, CXCL13, PPBP, CCL19*, and *CCL22)* cytokines *(IL21R, IFNG, IL12*, and *TNF)* immune system proteins *(CD27, ISG15, CD40L*, and *FASLG)* and skin-related interferon receptor repressor *USP18*. The downregulated genes include *IFNA5, CCL24, CCL27*, and *WIF1*, and *PPARG* using a log FC ≥−2 at a significance level of *P*_adj_ < 0.05. For the full list of complete upregulated vs. downregulated genes in canine CLE, (see Supplementary Table 2).

*CXCL10* (FC = 7.07), and *GZMB* (FC = 6.75) represented the highest fold upregulation, whereas *IFNA5* (FC = −5.7) represented the top down-regulated transcript. Neutrophil markers *S100A12* (FC = 6.53) and *CEACAM1* (FC = 1.82; canine ortholog of human *CEACAM3*) were highly expressed in CLE (Supplementary Table 2). T regulatory cell (Treg) gene *FOXP3* (FC = 4.12) and activated T cell markers *CD6* (FC = 3.93) and *CD27* (FC = 6.39) were also increased. To determine whether CLE with or without systemic symptoms (i.e., evidence of other organ involvement) could be ascertained based on chemokine expression, we plotted cell counts of *CXCL13* (overall FC = 5.8), a previously published biomarker of SLE, and found that it was more highly expressed in CLE with systemic symptoms than CLE without systemic symptoms (Figure 2C).

### CXCL10 Immunohistochemistry Reveals Immune Cells as the Main Source in CLE With Systemic Involvement vs. Keratinocytes in CLE Without SLE

We performed IHC to confirm protein-level expression of key CLE mediators (Figure 2D). CLE case 1 and case 2 expressed IFNγ and CXCL10 in infiltrating lymphocytes. CLE case 3 and case 4 slides, on the other hand, revealed significant perifollicular and epidermal staining of CXCL10, which appeared to originate from keratinocytes. Interface reaction patterns were remarkable at the dermal-epidermal junction in all four cases, with perivascular lymphocyte infiltration, as shown by the yellow star and red arrow.

### Cell Type Profiling Reveals Innate and Adaptive Immune Cell Infiltration of the Skin

**Figure 3:**
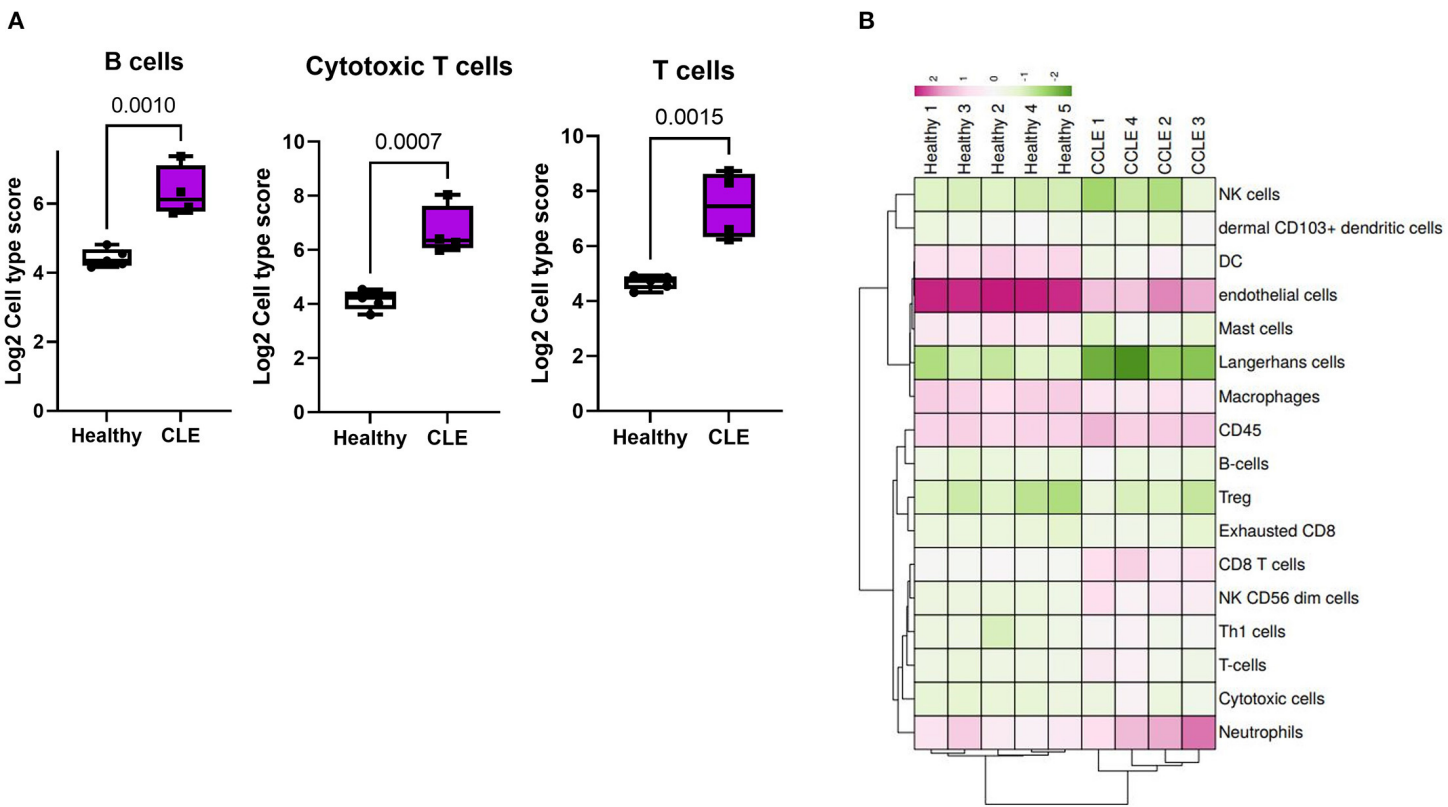
Cell type analysis in cases vs. controls. **(A)** B cell, cytotoxic T cell, and T cell enrichment in cases vs. controls based on NanoString advanced cell type analysis. **(B)** Heatmap of all cell type signature scores.

We determined the infiltration of various cell types in CLE lesional skin vs. controls by performing advanced cell type profiling using NanoString. *PTPRC* (gene that encodes CD45; FC = 2.01) was increased in CLE skin (Supplementary Table 2). Of those immune infiltrates, we could confidently predict the presence of B cells, T cells, and cytotoxic T cells based off of *p*-value cutoffs from advanced cell type profiling *p* < 0.05. All the cell types except Langerhans cells and macrophages differed significantly in CLE cases from controls, with significant increases in lymphoid populations in CLE skin (B cells *p* = 0.001; cytotoxic T cells *p* = 0.0007; T cells *p* = 0.0015; Figures 3A,B).

### Comparative Gene Expression Analysis of Canine, Human, and Mouse CLE Reveals Shared Inflammatory Gene Expression Signatures

**Figure 4:**
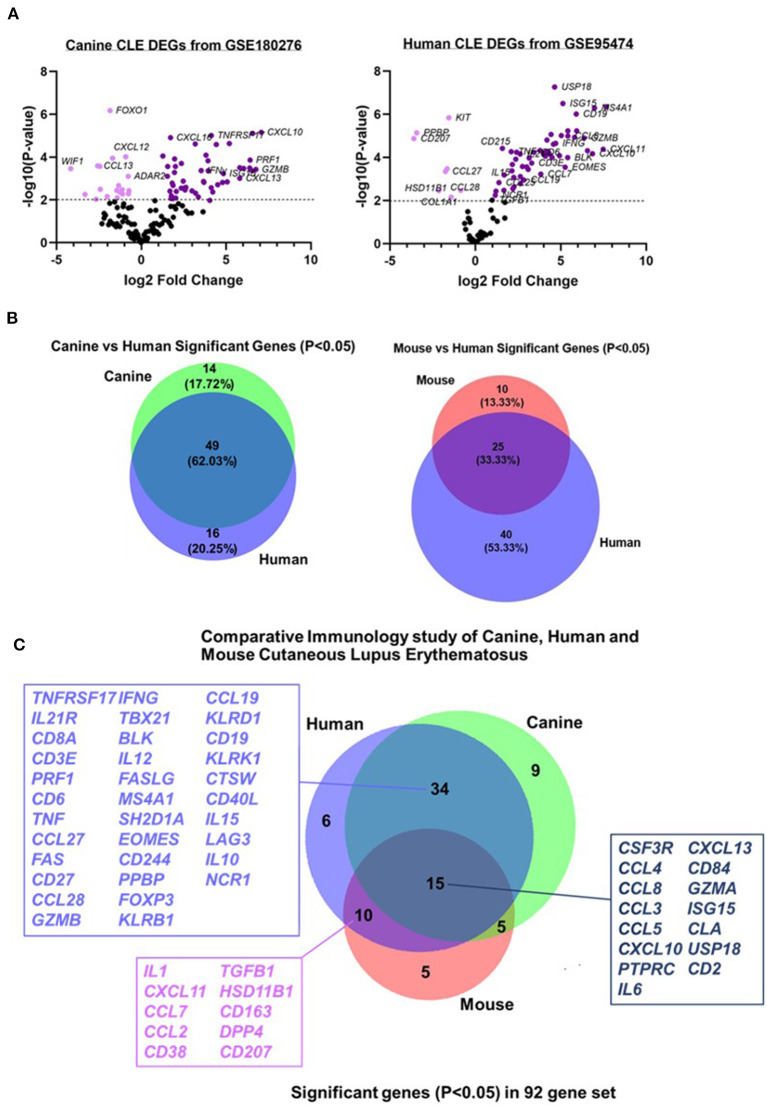
Comparative transcriptomics of canine CLE cases with human and murine CLE. Volcano plots of **(A)** canine and human CLE differentially expressed genes from 160 gene common denominator set. **(B)** Venn diagram showing number of shared probes between canine and human CLE. **(C)** Venn diagram showing shared DEGs between canine, human, and murine CLE.

To determine the conserved and shared factors of CLE immunopathogenesis, we compared our dog NanoString dataset to mouse (22) and human CLE microarray (GSE95474) datasets (23). First, we truncated the human dataset to match each of the corresponding datasets for canine and human CLE, so a common denominator was used for cross-species comparisons. We created canine and human volcano plots to examine overall up and downregulated genes in each species' dataset (Figure 4A). Next, we compared pairwise species expression of significant genes (examining *P* < 0.05 and *P* < 0.01 for CLE vs. healthy control). Comparison of canine and human revealed 62.03% overlap, mouse and human CLE revealed 33.33% overlap (Figure 4B) and overlap among the 92 significant gene probeset excluding four housekeeping genes (*B2M, RPL13A, CG14980*, and *HPRT*). Significant overlapping genes are listed in Figure 4C.

## Discussion

Like other autoimmune diseases, CLE can be difficult to diagnose, particularly when patients present with additional or atypical clinical features. Several tests, including inflammatory markers, cytokines and complements, antibodies and immunoglobulins, and histopathology, are evaluated in the event of an autoimmune disease to arrive at diagnoses. However, clinicians and physician-scientists still face a considerable challenge in correctly diagnosing and treating autoimmune diseases, including CLE (32). There is growing knowledge about the enormous opportunities that gene expression analysis provides for personalized medicine (33) and the efficiency of machine learning gene expression analysis in diagnosing autoimmune diseases (34–37).

All four dog cases from our study presented with CLE-like features in addition to other non-specific clinical signs, after which hierarchical gene expression clustering together with consultation from a veterinary pathologist and dermatologist helped clarify the diagnoses. Consistent with other published papers (23, 37, 38), our gene expression analysis of these four complex cases revealed high expression of *IFNG* and *CXCL10*, which are important biomarkers in CLE pathogenesis (39). Pro-inflammatory cytokines *IL12* and *TNF* were highly expressed in CLE, supporting the role of these genes in mediating lupus pathogenesis (40). There was no significant difference in the expression of pro-inflammatory cytokines *IL1* and *IL6* between canine CLE cases and healthy controls. This was not surprising because research studies that reported elevated levels of IL1 or IL6 in human CLE patients focused on subjects that had been pretreated with UV light or TLR2 agonists (41). Together with clinical and pathological findings, gene expression analysis of key mediators in these complex cases helped us arrive at a final consensus diagnosis of CLE. This approach of assigning transcriptional gene signatures based on the presence and absence of disease was used by Dey-Rao and Sinha to identify CLE disease drivers in human patients (42).

Chemokine (CXC motif) ligand CXCL10 is one of the highly expressed chemokines in CLE, thus making us curious about how this cytokine is expressed in skin-limited CLE vs. CLE with systemic involvement. In a prevalence study by Burge et al., mucosal lesions in SLE and CLE were distinct in both distribution and presentation. In contrast to oral ulcers in a significant portion of SLE patients, all CLE patients had chronic oral plaques with eyelid and nasal septum involvement (43). It is unclear whether the inflammatory cytokines identified in previous studies are expressed locally by the tissue or by the infiltrating immune cells in either clinical lupus subtype (44). The differences we observed in the origin of CXCL10 with immunohistochemistry in both skin-limited CLE and CLE with SLE may help distinguish between limited skin disease and systemic disease in dogs, warranting further studies in identifying specific methods for diagnosis. This would be particularly helpful for treatment decisions (45).

Our data also indicated expression level differences in *CXCL13* in CLE vs. CLE with systemic symptoms. Even though *CXCL13* is increased in skin-limited CLE compared to healthy skin, its role is still not well-understood. Some studies have suggested that CXCL13 is expressed as a result of epidermal damage *via* type 1 interferon signaling (46). Furthermore, our study supports the findings of Niederkorn et al., which suggests that CXCL13 is an activity marker for systemic but not cutaneous lupus erythematosus, authenticating its role in predicting systemic disease (46, 47). Based on these findings, we suspect the multiorgan involvement in case 1 (thrombocytopenia) and case 2 (elevated blood urea nitrogen indicative of kidney disease) may have been due to underlying SLE.

B cell gene signatures *CD19, TNFRSF17*, and *MS4A1* were significantly enriched in CLE skin tissue compared to healthy controls. High expression of NK cell signatures (*IL21R, KLRB1, KLRD1*, and *KLRK1*) and Cytotoxic T cell signatures (*CTSW, CD8A, EOMES*, and *LAG3*), as well as associated *GZMB* and *GZMA*, support previous CLE studies that described cytotoxic signatures (48–51). There was no difference in the expression of *IL4*, a Th2 cell gene signature, even though some studies have proposed the role of Th2 cells in the pathogenesis of CLE (52). There was a reduction in dendritic cell score and a significant increase in dermal CD103+ dendritic cell score similar to the structural alterations and loss of Langerhans cells in cutaneous lupus (53). Therefore, targeting specific pathways leading to their recruitment and activation in canine CLE could reduce inflammation and disease severity in patients (54).

Comparative analysis of canine CLE cases with human and mouse CLE revealed significant overlapping genes, supporting the conservation of inflammatory signatures across species. We previously published a similar frequency of DEG overlap in discoid lupus, which shares many DEGs with the complex CLE cases presented here (18). Of note, the clinical and histopathological features observed in CLE case 3 led us to consider the mucocutaneous variant of chronic CLE, as the dog experienced recurrent mucoulcerative lesions and a pattern of interface dermatitis mostly involving the hair follicles, similar to what has been previously observed in reported CLE cases (55). In this dog, we observed a 3-fold higher expression of *CCL23, CXCL8*, and *ISG15* compared with the other CLE cases; therefore, further studies could be performed to validate specific targets for CLE clinical subtypes in both canine and human patients.

We conclude that gene expression analysis can help researchers and clinicians better understand disease processes occurring in complex cases of autoimmunity. Lupus researchers have an opportunity to understand CLE and SLE immunopathogeneses using spontaneous animal models like canines since humans and canines share similar clinical characteristics and a considerable number of DEGs. Induced animal models used to study lupus have provided some clues on the role of immune factors in various organs, including the skin, and have guided the development of some treatments to manage the cutaneous manifestations of lupus. Our study and previous research studies show that using gene expression analysis, clinical and pathological features of spontaneous autoimmune diseases in dogs will be key to fully understanding disease pathogenesis and bridging the gaps with mouse models and human medicine while also providing useful treatments for veterinary patients.

## Data Availability Statement

The canine dataset presented in this study can be found on Gene Expression Omnibus (GEO) Database under Accession GSE180276.

## Ethics Statement

The animal study was reviewed and approved by Cummings School of Veterinary Medicine, Tufts, IACUC. Written informed consent was obtained from the owners for the participation of their animals in this study.

## Author Contributions

JR: conceptualization, project administration, and funding acquisition. NR and JR: methodology and resources. CD: software. RA, CP-M, and JR: validation. AA, DM, NW, and JR: formal analysis. CP-M, NR, JR, and CD: investigation. NR, AA, CP-M, RA, and JR: data curation. AA: writing—original draft. AA, CP-M, and JR: visualization. JR, RA, and NR: supervision. All authors: writing—review and editing. All authors contributed to the article and approved the submitted version.

## Funding

JR was supported by a Career Development Award from the Dermatology Foundation and a Target Identification in Lupus award from the Lupus Research Alliance.

## Author Disclaimer

The views presented are the authors and do not reflect the opinions of the funders.

## Conflict of Interest

JR is an inventor on patent application #15/851,651, “Anti-human CXCR3 antibodies for the Treatment of Vitiligo,” which covers targeting CXCR3 for the treatment of vitiligo; and on patent #62489191, “Diagnosis and Treatment of Vitiligo” which covers targeting IL-15 and Trm for the treatment of vitiligo. NR is an employee of Alnylam Pharmaceuticals. CD is an employee of NanoString Technologies. The remaining authors declare that the research was conducted in the absence of any commercial or financial relationships that could be construed as a potential conflict of interest.

## Publisher's Note

All claims expressed in this article are solely those of the authors and do not necessarily represent those of their affiliated organizations, or those of the publisher, the editors and the reviewers. Any product that may be evaluated in this article, or claim that may be made by its manufacturer, is not guaranteed or endorsed by the publisher.

## Supplementary Material

Supplementary Figure 1 Examination of gene expression signatures in cases compared to well-defined veterinary patient gene signatures. **(A)** Hierarchical cluster analysis of 160 lupus-associated genes across the four cases presented here, seven dogs with DLE, one dog with suspected MLE, four dogs with pemphigus entities, and five healthy controls. **(B)** Principal Component Analysis (PCA) displays clear spatial separation of healthy margin controls; however, CLE, DLE, and pemphigus entities share similar gene expression patterns.

Supplementary Table 1 Probe set and annotations.

Supplementary Table 2 Comparison of human and canine datasets.

Supplementary Table 3 Complete DEG list.

Supplementary Document 1 Additional veterinary care information.

## Abbreviations

FC: Fold change
H&E: hematoxylin and Eosin
CLE: cutaneous lupus erythematosus
SLE: systemic lupus erythematosus
FFPE: formalin fixed paraffin embedded.
