# Supplementary figures and images for: Using Gene Expression Analysis to Understand Complex Autoimmune Skin Disease Patients: A Series of Four Canine Cutaneous Lupus Erythematosus Cases
Source: Front Vet Sci. 2022 Feb 24;9:778934. doi: 10.3389/fvets.2022.778934 (PMC8907585; doi:10.3389/fvets.2022.778934)

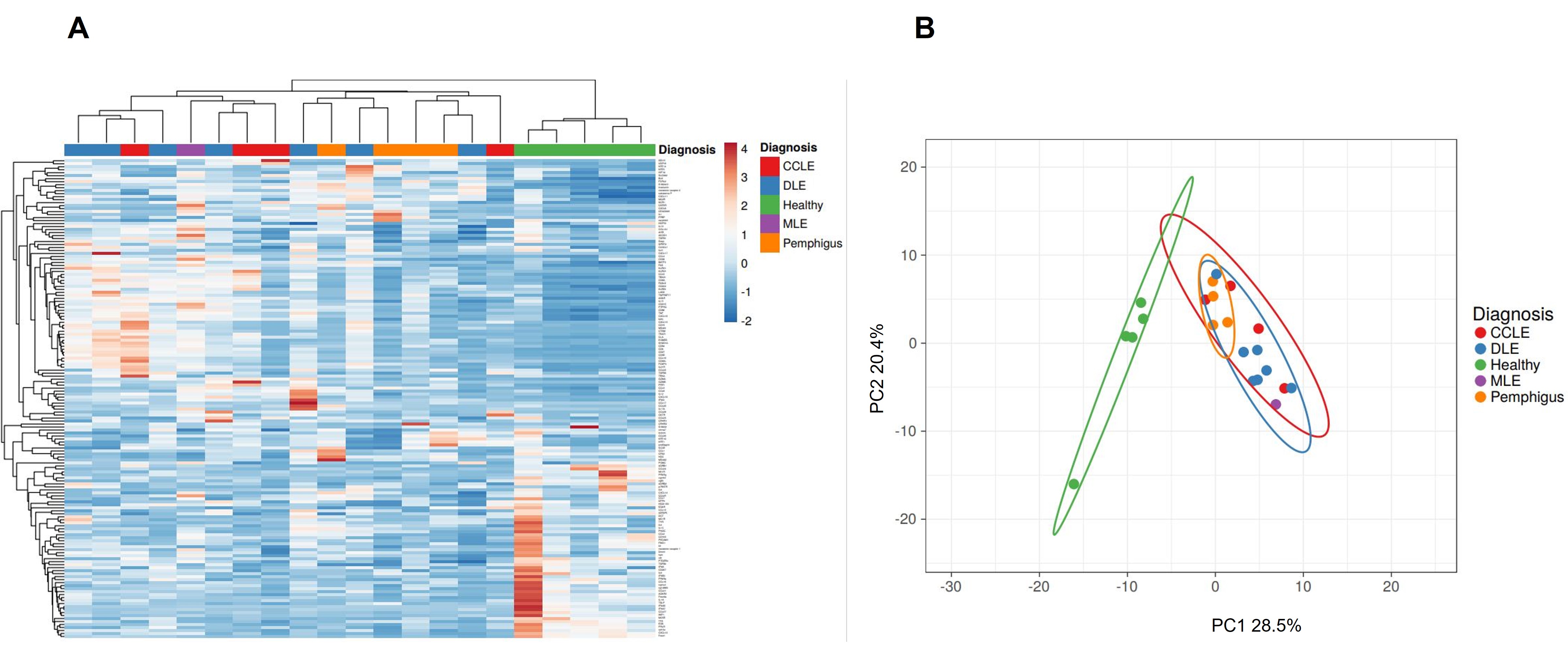

Supplement: Supplementary Figure 1 — Examination of gene expression signatures in cases compared to well-defined veterinary patient gene signatures. (A) Hierarchical cluster analysis of 160 lupus-associated genes across the four cases presented here, seven dogs with DLE, one dog with suspected MLE, four dogs with pemphigus entities, and five healthy controls. (B) Principal Component Analysis (PCA) displays clear spatial separation of healthy margin controls; however, CLE, DLE, and pemphigus entities share similar gene expression patterns. [file Image_1.JPEG]
