# Supplementary material for: Using Gene Expression Analysis to Understand Complex Autoimmune Skin Disease Patients: A Series of Four Canine Cutaneous Lupus Erythematosus Cases
Source: Front Vet Sci. 2022 Feb 24;9:778934. doi: 10.3389/fvets.2022.778934 (PMC8907585; doi:10.3389/fvets.2022.778934)
Supplement: Supplementary Document 1 — Additional veterinary care information. [file Data_Sheet_1.docx]

**CLE Case 1**

**Visit 1**

[A] 7-year-old CM GSD was presented for a one-year history of progressive superficial crusting lesions along the dorsal aspect of the thoracolumbar spine. He has previously been treated with numerous courses of antibiotics, all for about 10-14 days: cephalexin 100)mg BID, cephalexin 750mg TID, Clavamox 500mg BID, Simplicef 300mg SID, doxycycline 200mg BID, and marbofloxacin 100mg SID.

Physical examination revealed clumps of crusted hair adherent to the skin along the thoracolumbar region bilaterally over the dorsum. He also had a focal 1cm alopecic, eroded lesions on the right foreleg just proximal to the carpus consistent with an acral lick granuloma. Superficial cytology (tape test) revealed one cluster of cocci, consistent superficial pyoderma. We performed an aerobic culture and susceptibility today and will prescribe antibiotics accordingly at the beginning of next week. As the owner also notes polyphagia and behavioral change (increased affection) at home, we also performed a CBC/CHEM/T4 to rule out a primary endocrinopathy.

Thank you for the courtesy of your referral. If you have any questions or concerns regarding the management of this case please do not hesitate to give us a call.

**Visit 2**

[A] 7.5yo CM German Shepherd, was presented on 1/22/13 for further workup of his documented thrombocytopenia, as well as to see the Dermatology service for skin biopsy of a lesion over his caudodorsum unresponsive to antibiotics. We discussed primary and secondary thrombocytopenia, and causes for both. We also discussed our recommended work-up, consisting of platelet count, PT/PTT, tick titers with Lyme C6, abdominal ultrasound, and three-view thoracic radiographs. The owners declined any imaging, for multiple reasons, with the most pertinent reason being they would not pursue any treatment if neoplasia was found. We discussed with owners that these tests were necessary to complete a full work-up and determine the most likely cause of his thrombocytopenia, and with those results, we would best be able to determine the most appropriate treatment regimen. The owners still declined, and only elected to perform the recommended bloodwork and skin biopsy with Dermatology at this time.

We discussed the fact that the owners are very adverse to the idea of treating with corticosteroids due to [his] poor response to his last course. We discussed azathioprine as another treatment option that we usually use in combination with corticosteroids, but that we could use alone as well. We discussed side effects, such as bone marrow suppression and greater susceptibility to infection, and the need for frequent CBC and platelet counts during the initiation phase of the treatment to make sure that [he] was handling the medication appropriately and was responding. The owners seem open to this idea, and plan to research azathioprine further before making a decision. They voiced concern over any possibility of side effect with treatment. Something not discussed with [his] owners, but something that other medicine clinicians have used with some success here is melatonin, which may be something to consider if the owners are too concerned about the side effects of azathioprine.

At the time of discharge, the results of the bloodwork were not yet back. The platelet count today was 6 K/uL, and PT/PTT was within normal limits. (PT 7, ref 6.2-9.3; PTT 10.2; ref 8.9-16.3). The tick titers will be back early next week.

The Dermatology service performed the skin biopsy under dexmedetomidine sedation, and submitted the samples for histopathology. These results will be back next week.

INTERNAL MEDICINE: Thank you for bringing [him] in to see [our] Department. [He] had thrombocytopenia (low platelet count) that has been documented since August of 2012. He has been asymptomatic for this low platelet count with no episodes of bloody or tarry stools, no episodic lethargy, and no episodes of collapse. However, at [his] most recent appointment with our Dermatology service his platelet count was only 13,000/uL which is low enough that we are concerned about spontaneous bleeding. We discussed that there are several causes of thrombocytopenia. Primary or idiopathic thrombocytopenia is when the immune system destroys platelets for no reason that we are able to find. Secondary thrombocytopenia is caused by many things which incite the immune system to attack the platelets. Documented causes of secondary thrombocytopenia are tick-borne diseases, cancer, various drugs, and vaccine reactions. Primary thrombocytopenia is a diagnosis of exclusion, meaning that we can only truly diagnose a patient with this disease when we have ruled out all other reasonable causes of secondary thrombocytopenia.

To rule out causes of secondary thrombocytopenia we recommended more extensive tick titer panels than have been performed previously, and an abdominal ultrasound and 3 view chest x-rays to rule out gross evidence of cancer. Then to make sure that [his] existing platelets are functioning properly we recommended coagulation tests. And finally, to monitor where [his] platelet levels are today we recommended rechecking the platelet count.

You elected to proceed with the tick titers, coagulation function testing, and the platelet count but declined to pursue an abdominal ultrasound or chest x-rays. We will call you with the results of the coagulation function tests and the platelet count tonight. The tick titers will come back in 3-5 business days so we will call you next week when we have these results available.

Once we have all of our test results, including the skin biopsy results, we will discuss the appropriate next course of therapy. Today we discussed azathioprine, an immunosuppressive drug which is the second line of drugs to treat idiopathic thrombocytopenia (since [he] had a poor reaction to prednisone previously). We talked about the fact that if [he] goes on this drug he will need frequent monitoring of the blood cell levels (about every 2 weeks initially) but this could be done at your local veterinarian. The goal of azathioprine therapy is to suppress the immune system enough that the body can return platelet levels to normal. Most patients can then have the dose decreased and eventually stopped. However, 20-50% of patients will experience a relapse or reoccurrence of the thrombocytopenia. The time to relapse can range from 1-6 years after stopping therapy.

**>>There are no further medical records, lab results, notes or client communication<<**

**CLE Case 1 biopsy report notes:**

“…[T]hese sections contained a lupus-like reaction pattern and would consider it to best fit into the category of “chronic cutaneous lupus.”

Microscopic findings:

Haired skin: Diffusely, there is a lichenoid band of lymphocytes, plasma cells, and macrophages that often blurs the dermal-epidermal junction as well as the dermal-follicular junction with frequent loss or obscuring of the basal cell layer. Remaining basal cells are frequently vacuolated. Occasional vacuolation of suprabasal cells is seen. There is frequent pigmentary incontinence. In some areas, there is mild fibrosis of the superficial dermis. Sebaceous glands are not seen. There is diffuse, mild, regular epidermal hyperplasia with moderate hyperkeratosis that contains segments of parakeratosis (however, a granular cell layer is typically present). Minimal, perivascular lymphoplasmacytic infiltrates are present in the deeper dermis.

**CLE Case 2**

**Visit 1**

Problem: History of skin lesions, fever, shifting leg lameness

Report:

[He] was brought into the internal medicine service for evaluation after an approximate 1-week history of lethargy and decreased appetite. He also has an approximate 5-year history of crusty skin lesions (diagnosed as lupus by his regular veterinarian but not treated) and a 2-day history of apparent shifting leg lameness and fever. He was treated by his veterinarian with Baytril and Prednisone for the last 2 days. This resulted in a decreased body temperature and resolution of the lameness.

Today, [he] is quieter than normal but alert and responsive with normal body temperature. His physical examination was unremarkable aside from thick crusty skin lesions over his muzzle, around his eyes, at his inner proximal pinnae (ears), and a patch of shaved skin on his back. His fur had been shaved for aspiration of a mass that was interpreted as benign by his veterinarian a couple of weeks ago.

As we discussed, there are numerous potential causes for [his] fever, lethargy, and decreased appetite. We took some blood and urine for general health screening and chest x-rays to rule out cardiac or lung disease as a component of [his] fever and lethargy. X-rays showed normal cardiac and pulmonary structures without evidence of cancer or other abnormalities.

In order to diagnose the disease of his joints and/or skin, we need to obtain joint fluid and a skin biopsy. Unfortunately, prednisone will hamper interpretation of these tests due to its immunosuppressive actions. For now, we are going to stop the steroids and treat empirically with doxycycline in case an infectious disease such as Lyme (negative test before could have been taken before [he] made antibodies in response to disease exposure) could be causing his clinical signs. If his fever and lameness return once he is off steroids, we strongly recommend the diagnostic tests described above.

Please call tomorrow for the blood and urine test results if you do not hear from us tonight.

Please give the Doxycycline as directed. Please continue the Baytril as previously prescribed but stop administering the prednisone.

Doxycycline

100mg

28

Give 1 tablet by mouth with food twice daily for 14 days.

**Visit 2**

Problem: History of skin lesions, fever, shifting leg lameness

Report:

[A] 10-year-old Mixed-breed dog, was seen at our institution 5/19 for evaluation of an approximate 1-week history of lethargy, decreased appetite, and a 2-day history of shifting leg lameness and fever. The lameness and fever had responded to antibiotic and steroid treatment by his regular veterinarian. He has an approximate 5-year history of crusty skin lesions (diagnosed as lupus by his regular veterinarian but not treated). After a physical examination, chest x-rays, and bloodwork, and urinalysis were taken, [he] was sent home on empirical doxycycline therapy and his steroids were stopped. It was recommended that [he] have skin biopsies and/or samples of joint fluid taken in order to diagnose the source of his clinical signs.

After initially doing well at home, [he] had increased lethargy, reluctance to walk, and recurrence of his fever on 5/25 and was dropped off today for a recheck examination, blood work, and skin biopsy.

On physical exam, [he] was alert and responsive with a body temperature of 103.5. He continues to have thick crusty skin lesions over his muzzle, around his eyes, at his inner proximal pinnae (ears), and on his back. He also has peeling and separation on the pads of his feet, along with some sites of infection, which contained small amounts of pus. It is likely that at least part of [his] episodes of lameness is due to pain from the infection and peeling of his footpads.

We repeated [his] blood work and found that he continues to have an increased ALP (one of his liver enzymes) level despite being taken off Prednisone. As was discussed, we have submitted an ACTH stimulation test to investigate the possibility that [he] may have Cushing’s Disease. We were unable to fit an abdominal ultrasound in today but would like to schedule one to evaluate his liver and adrenal glands.

Skin biopsies were taken today while under sedation. Small samples were taken from his back, right ear, nose, and left front footpad. He has sutures at the biopsy sites on his back, ear, and nose, which should be removed in 10-14 days.

As it appears that [his] feet have pockets of infection, we will be sending him home on some antibiotics, as well as an anti-inflammatory to keep him comfortable while his biopsy sites and feet heal. It is our hope that the results of the skin biopsies, blood tests, and ultrasound will help us determine the source of [his] skin lesions. Based on their findings, we will reassess his future treatment.

[His] biopsy results and ACTH test are pending and should be in either tomorrow or early next week. Please call us tomorrow for an update. In addition, as we were unable to schedule an appointment for an abdominal ultrasound this week, we should also discuss scheduling an appointment for next week as well.

Please give the medications as directed below. If [he] experiences vomiting or diarrhea please stop the medications and give us a call.

Please monitor biopsy sites for any signs of increased redness, swelling, or exudate, which could indicate infection, and have him evaluated promptly. The sutures can be removed in 14 days and can be done either here or at your regular veterinarian.

Please stop administering the Rimadyl given to you by your veterinarian but continue the doxycycline until it is gone.

Deramaxx

25mg

5

Give 1 tablet by mouth once daily with food.

Cephalexin

500mg

42

Give 1 tablet by mouth twice daily for 3 weeks.

Chlorhexiderm flush

12 oz

1 bottle

Soak feet in a bowl twice daily for 5 minutes for 7 days.

**Visit 2 follow up with primary veterinarian:**

[He] came in yesterday for evaluation of his skin disease and recurrent lameness. Repeat CBC and chemistry profiles were drawn. These were unremarkable aside from substantially elevated SAP. We ran an ACTH stimulation test, which came back in the normal range. Skin biopsies were diagnostic for pemphigus foliaceous.

[He] was sent home yesterday with a 3-week course of Cephalexin and we are adding these additional treatments:

Chlorhexidine scrub: soak all feet for 5 minutes twice daily for the next 5 days.

Azathioprine 50mg (# 30): Give 1 tablet by mouth once daily for 7 days. Then administer once every other day.

Prednisone 20mg (#60) Give 1 tablet by mouth twice daily for 2 weeks. Then give 1 tablet by mouth once daily for 2 weeks. We will wean the prednisone further based on how [his] skin is doing at that time.

We recommend a recheck exam in 7-10 days to assess response and monthly CBCs for the first 3 months of treatment to monitor [him] for bone marrow suppression secondary to Azathioprine administration.

**Visit 3**

Problem: Pemphigus foliaceous

Report:

[He] was diagnosed with pemphigus foliaceus 3-4 weeks ago and has since been treated with azathioprine, prednisone, and cephalexin. [He] has improved well to therapy thus far but still has a little way to go. Today, his physical exam revealed a great improvement in the skin lesions of his front legs, nose, over his eyes, and feet. The area on his back is much less crusty but is itchy, reddened in spots, and seems to bother [him]. He is otherwise bright and alert and is doing very well at home according to his mom. Today, we removed his biopsy site stitches and drew blood for CBC and liver profile. These results should be available tomorrow afternoon.

Please put a t-shirt on [him] to protect his back lesion from the sun.

Please call tomorrow afternoon for blood work results.

Please schedule an appointment in about 1-month for a recheck examination CBC. You can do this with either your regular veterinarian or at our institution. If [his] condition starts worsening once more, please have him re-evaluated sooner.

Azathioprine

50mg

Owner has

Give 1 tablet by mouth once every other day.

Cephalexin

500mg

Owner has

Give 1 tablet by mouth twice daily until gone.

Chlorhexiderm flush

12 oz

1 bottle

Soak lesions on back and feet once every other day for 5 minutes.

Prednisone

20mg

Owner has

Give 1 tablet by mouth once every other day for the next 2 weeks. Then give ½ tablet once every other day.

**Visit 4:**

History of pemphigus foliaceus ruptured mass over the dorsum.

IMAGING FINDINGS

THORAX, THREE VIEWS: The cardiovascular structures are within normal limits. Within the left cranial lung lobe, there is a patchy increase in soft-tissue opacity and there is a leftward shift of the cardiac silhouette; this is thought to be due to partial atelectasis secondary to general anesthesia. The pulmonary parenchyma is otherwise within normal limits. An endotracheal tube is present with the tip at the level of C6. There is air within the cervical and intrathoracic esophagus. There is an increase in soft tissue opacity within the cranial abdomen. Over the thoracolumbar region, the dorsal soft tissues are markedly irregular and heterogeneous. Within the dorsal subcutaneous tissues superimposed over the scapulae, there is a 9mm x 8mm mineral opacity. There is cervical spondylosis deformans.

IMAGING CONCLUSIONS

Normal thorax. No evidence of pulmonary metastatic disease. Increased soft-tissue opacity within the cranial abdomen is thought to represent hepatomegaly. An abdominal ultrasound can be considered to further evaluate this finding. The irregularity of the subcutaneous tissues of the thoracolumbar region is consistent with the clinically reported ruptured mass. Mineralized subcutaneous nodule.

DIAGNOSES

Adverse events: episode of gastric dilatation

Provisional diagnoses :

Recommendations: see below

Outcomes :

No evidence of :

Differential diagnoses : abscess/chronic infection vs neoplasia (cancer) vs immune-mediated disease

Procedures: blood work (blood gas analysis, complete blood count, chemistry), urinalysis, imaging (thoracic and abdominal radiographs), dermatology consultation with skin biopsies (nose and mass), surgery with mass removal and biopsy, grenade closed suction drains placed, orogastric tube passed to decompress stomach on 3/3/13

Final diagnoses: post-operative ulcerated and infected mass removed from dorsum, episode of gastric dilatation

History and Physical Exam Summary:

[A] 16-year-old mixed breed was presented for a rupture of a mass on her dorsum, that ruptured overnight prior to presentation. Previously, a mass was removed from this area in 2004 but owners report that the area had never completely healed. The owners report that he had been doing well at home prior to this. No v/d/c/s. Previously diagnosed with Pemphigus 9 years ago. Not currently being treated for this disease. Current meds are HW/flea/tick prevention only, described as having good energy and behaving like a puppy prior to this incident. He hasn't lost an appreciable amount of weight according to the owner but never seems to keep on weight either. Later in the day, rDVM records indicated he was seen last summer (6/12), and the mass on the dorsum was aspirated and came back as suspect basilar cell epithelial tumor (basilar cell carcinoma), but pemphigus could not entirely be ruled out. There is always irregular tissue over the dorsum and he licks at it incessantly. It was removed once several years ago and has never healed, but has also never been ruptured open as it was on admit. [He] is described to be eating/drinking/urinating/defecating normally.

T: 100.6 HR: 120/min RR: 32/ no eff MM: pink/black tacky CRT:&amp;lt; 2sec

EENT: lenticular sclerosis OU, mild ocular discharge, marked dental calculus, and dental disease.

PLNS: Right prescapular LN palpably enlarged (~2cmx 1.5cm lobulated, firm). other PLNs WNL

H/L: NMA, pulses fair. normal bronchovesicular sounds heard bi-laterally

MSI: BCS 2.5/9, MCS: 1.5/9, approx 5x6 cm irregular ulcerated area on dorsal region (thoracolumbar area). Several pinpoint to 5m round firm nodules on the dorsum of mussel. Ulcerated lesions on nasal planum bilaterally. focal ulcerated lesion on LF footpad, soft SQ mass medially over left antebrachium

ABD: Soft on palpation, no pain elicited, no palpable masses.

UG: normal external CM urination

NEURO: appropriate mentation, ambulatory x4

A1: Ulcerated mass on dorsum r/o neoplasia (SSC vs lymphoma vs other) vs abscess vs benign non-healing wound

A2: Pemphigus Foliaceus

A3: Weight loss r/o endocrine disease vs metabolic vs dietary vs infectious vs paraneoplastic.

A4: Anemia r/o chronic inflammation vs paraneoplastic vs kidney disease vs GI loss vs primary bone marrow vs iron deficiency/dietary

A5: Azotemia r/o Pre-renal vs renal vs post renal

A6: Hyperlactatemia R/O tissue damage/necrosis (ulcerated mass) vs dehydration/decreased tissue perfusion

A7: Elevated ALP R/O hepatobiliary disease vs endocrine disease (Cushings dz) vs

A8: Elevated ALT R/O hepatocellular damage (ischemia vs neoplasia)

P1: Bx nasal planum, FNA Prescapular LN, impression smear of ulcerated mass.

P2: 3 view CXR

P3: Soft tissue resection of ulcerated mass w/ histopathology

Patient Condition: stable

Initial and Current Therapy:

IVF @ 50mls/hr

Fentanyl CRI @ 3mcg/kg/hr

Biopsy procedure:

Midazolam 3.5mg IV pre-med

Propofol 6ml induction/ maintenance

Intubated and maintained on Isoflurane

Diagnostic tests completed and pending:

NOVA: Lac: 4.4 BUN:34 PCV: 27 TS: 5.3

Chem: BUN:37, ALT: 98, ALP:581, TP: 5.3

CBC: HCT: 24

Thoracic radiographs: No evidence of metastatic disease. See full report

FNA of right prescapular LN - fatty material

Blood Type: DEA 1.1

Saline Agglutination: negative

Procedure: Impression smears of ulcerated area, nasal biopsy

(Midazolam 3.5mg IV pre-med

Propofol 6ml induction/ maintenance

Intubated and maintained on Isoflurane)

FNA: Right prescapular LN

Additional Requests Submitted:

BX of mass pending

Preliminary Diagnostic Results:

see problem list above

Prognosis Given Owner: strongly suspect malignant neoplasia, but a biopsy will guide further prognosis, for now, will focus on surgery, recovery, and infection oxygen

Client and Referring Veterinarian Communication Status: see comm log

S: Pain well controlled overnight, some trouble going for walks overnight (reluctant to rise/walk) but much brighter this morning. Received a total of 1/2 unit pRBCs intra-op and post-op. PCV/TS stable since blood transfusion. Ate treats for owner but refusing proper dog food. Drinking on own. Overall stable, and good/restful day.

O: TPR stable all day, MM-pk/moist CRT &amp;lt;2sec

EENT: lenticular sclerosis OU, unchanged ulcerative lesions on nares (no active oozing), marked dental calculus, and severe periodontal disease.

PLNS: R. pre-scapular LN enlarged (lipoma vs true lymphadenopathy), others WNL

H/L: NMA, pulses strong/synchronous, normal bronchovesicular sounds heard bi-laterally, eupneic

MSI: BCS 2.5/9, MCS: 1.5/9, incision over dorsum extends mid-scapula along midline to approximately level of L3-4 looks good, Tegaderm changed and lidocaine patch placed laterally over either side of the suture line, 2 grenade closed-suction drains in place producing approximately 1-2 ml/hour total dark red serosanguineous fluid, unchanged lesions over the dorsal nose, a single suture in place from punch skin biopsy site, soft SQ mass medially over left antebrachium unchanged

ABD: Soft on palpation, no pain elicited, no nausea, rectal WNL with formed stool palpated

UG: normal urinations

NEURO: appropriate mentation though somewhat sedate on fentanyl CRI, ambulatory x4, full exam not performed but no obvious neurologic deficits

IVC: Ceph. 20 sterile jelco (day 2), anesthesia IVC removed overnight

Nutrition: eating treats consistently, not eating dog food when offered, drinking well

Diagnostics:

Big 4/lactate: stable with improved but persistent hyperlactatemia

A1: 1d post-op ulcerated/ruptured/infected deep dermal mass removal dorsum r/o neoplasia (carcinoma vs lymphoma vs other) vs chronic abscess (r/o atypical infection-nocardia vs mycobacterium) vs immune-mediated (pemphigus)

A2: Historic Pemphigus Foliaceus

A3: Cachexia r/o paraneoplastic vs endocrine disease vs protein-losing disease vs dietary deficiency vs cardiac cachexia (little evidence of this)

A4: Anemia (normocytic, normochromic): stable post-transfusion r/o rebounding from acute blood loss from the day prior vs chronic inflammation vs paraneoplastic vs kidney disease vs GI loss vs primary bone marrow vs iron deficiency/dietary

A5: Mildly elevated BUN with normal creatinine (did not re-check post-op, Azo stick has been stable): r/o pre-renal vs renal (CKD with creatinine low due to cachexia/muscle wasting) vs GI bleed

A6: Hyperlactatemia (improved, but persistent) R/O Type A perfusion derangement (blood loss vs dehydration) vs Type B (neoplasia vs hepatic disease vs mitochondrial dysfunction from infection/illness)

A7: Elevated Liver Enzymes (moderate, mixed pattern, primarily induction enzymes) R/O hepatobiliary disease (neoplasia vs hepatitis/chronic hepatopathy) vs endocrine disease (Cushings dz) vs hepatocellular damage (ischemia vs neoplasia) vs benign aging change

A8: thrombocytosis: r/o Cushing’s disease vs GI bleed

P1: continue pain meds (fentanyl CRI with plan to taper overnight, lidocaine patch replaced), antibiotics (IV unasyn), biopsy pending, and plan for follow-up/oncology consult to be made based on results

P2: biopsy of dorsal nasal lesions pending, not currently on medications for this disease

P3: consider nutrition consult if weight loss/lack of weight gain is a problem

P4-8: re-check values as needed based on clinical signs, consider AUS when healed from surgery, consider starting Pepcid at home in case of GI bleed

CASE PROGRESS 3/3/13

S: BAR, continued to eat treats well overnight but no food. Pain well controlled when fentanyl CRI tapered and discontinued. Grenade drains were both removed this morning due to minimal production. Had an event on a walk with owners around 11 am where he got worked up, was hypersalivating, retching, and appeared gas distended. An abdominal x-ray (right lateral) revealed gastric dilatation with partial/early rotation. An orogastric tube was passed for gastric decompression. A post-procedure radiograph confirmed stomach/pylorus in correct position, but still significant amount of ingesta in the stomach. Very stable afterwards and taking oral medications well (See below for details).

O: TRP stable all day (apart from gastric dilatation episode where she was tachypneic and mildly tachycardic for a bit. MM-pk/moist CRT &amp;lt;2sec

EENT: lenticular sclerosis OU, unchanged ulcerative lesions on nares unchanged, marked dental calculus, and severe periodontal disease.

PLNS: R. pre-scapular LN enlarged (lipoma vs true lymphadenopathy), others WNL

H/L: NMA, pulses strong/synchronous, normal bronchovesicular sounds heard bi-laterally, eupnic apart from tachypnea during gastric dilatation event

MSI: BCS 2.5/9, MCS: 1.5/9, incision appears to be healing well so far, comfortable on light palpation, Tegaderm removed and lidocaine patch reapplied today, grenade drains removed this morning, unchanged lesions over the dorsal nose, a single suture in place from punch skin biopsy site, soft SQ mass medially over left antebrachium unchanged

ABD: Soft on palpation, in the morning, became markedly tympanic and distended during bloat event, then resolved post-gastric decompression, ptyalism and retching were severe during the event but resolved after recovery from anesthesia, formed stool noted today

UG: normal urinations

NEURO: appropriate mentation/BAR, ambulatory x4, full exam not performed but no obvious neurologic deficits

IVC: Ceph. 20 sterile jelco (day 3)

Nutrition: eating treats consistently, not eating dog food when offered, drinks well, encouraging small meatballs post-bloat event to promote GI motility

Diagnostics:

Big 4/lactate 8am: PCV: 32%, TS 6.3, Azo 15-26, lac 3.1, BG 109

Big 4/lac post-gastric decompression: PCV: 28%, TS 6.0, Azo 30-40, BG 166, Lac 1.9

Lat R. AXR: marked gastric dilatation with early pyloric rotation/dorsal displacement

Lat R. AXR post-gastric decompression: gastric positioning is normal, a moderate amount of ingesta in the stomach, some gas dilated loops of intestine noted (final radiology review pending)

UA (free catch, submitted 3/2/13): USG 1.010 on IVF, 20-30 WBCs/hpf, trace bilirubin crystals

Procedure:

Under general anesthesia with acepromazine as pre-med, and propofol IV for induction, an ET tube was placed and an orogastric tube passed for gastric decompression. A large amount of air was removed, only a tiny amount of liquid ingesta was removed. The stomach markedly decreased in size. No complications during procedure or recovery. The entire procedure from induction to extubation was approximately 20 minutes.

A1: 2d post-op ulcerated/ruptured/infected deep dermal mass removal dorsum r/o neoplasia (carcinoma vs lymphoma vs other) vs chronic abscess (r/o atypical infection-nocardia vs mycobacterium) vs immune-mediated (pemphigus)

A2: Historic Pemphigus Foliaceus

A3: Cachexia r/o paraneoplastic vs endocrine disease vs protein-losing disease vs dietary deficiency vs cardiac cachexia (little evidence of this)

A4: Anemia (normocytic, normochromic): stable r/o rebounding from acute blood loss from admit vs component of dilution vs chronic inflammation vs paraneoplastic vs kidney disease vs GI loss vs primary bone marrow vs iron deficiency/dietary

A5: Mildly elevated BUN with normal creatinine (did not re-check post-op, Azo stick has been stable, with sl. elevation this afternoon): r/o pre-renal vs renal (CKD with creatinine low due to cachexia/muscle wasting) vs GI bleed

A6: Hyperlactatemia (improving) R/O Type A perfusion derangement (blood loss vs dehydration) vs Type B (neoplasia vs hepatic disease vs mitochondrial dysfunction from infection/illness)

A7: Elevated Liver Enzymes (moderate, mixed pattern, primarily induction enzymes) R/O hepatobiliary disease (neoplasia vs hepatitis/chronic hepatopathy) vs endocrine disease (Cushings dz) vs hepatocellular damage (ischemia vs neoplasia) vs benign aging change

A8: thrombocytosis: r/o Cushing’s disease vs GI bleed

A9: Gastric dilatation: r/o GI motility disorder vs anxiety/excitement induced

P1: fentanyl CRI discontinued and transitioned to oral tramadol, lidocaine patch replaced, antibiotics (IV unasyn, TGH on oral Clavamox), biopsy pending and plan for follow-up/oncology consult to be made based on results, suture removal in 10-14 days

P2: biopsy of dorsal nasal lesions pending, not currently on medications for this disease

P3: consider nutrition consult if weight loss/lack of weight gain is a problem

P4-8: re-check values as needed based on clinical signs, consider AUS when healed from surgery, start Pepcid at home in case of GI bleed

P8: Cerenia given during gastric decompression, metoclopramide CRI 2mg/kg/d until discharge, home with instruction to watch closely for recurrent signs

PROFESSIONAL REPORT

[He] was presented to our institution due to a large mass over the dorsum that was infected and had ruptured. He was hospitalized for care and diagnostics. On 3/2, after consulting with the dermatology and surgery services, the decision was made for to remove the mass in order to control the infection and submit for biopsy. The dermatologist also took a punch biopsy of the nose to rule out active pemphigus lesions, given the history of pemphigus foliaceus diagnosed in 2004. [He] had not been on any medications in years for this, and currently was only on heartworm/flea/tick prevention. The owners described him as acting puppy-like prior to this event. He hasn't lost an appreciable amount of weight according to the owner, but never seems to keep on weight either, and has a cachexic appearance. Referring veterinarian records were faxed to the ER and indicated he was seen last summer (6/12) and the mass on the dorsum was aspirated and came back as suspect basilar cell epithelial tumor (basilar cell carcinoma), but pemphigus could not entirely be ruled out. The owners report there always irregular tissue over the dorsum and he licks at it incessantly. It was removed once several years ago and has never healed, but has also never been ruptured open as it was on admit, and they had not appreciated the infection before this.

Pre-operative blood work revealed anemia consistent with acute blood loss, and stable moderate mixed liver enzyme elevations (primarily ALP changes, mild ALT activity, normal tbili). He had persistent hyperlactemia that improved but never resolved despite cardiovascular stabilization during his stay. This likely represents a type B hyperlactemia (i.e.. secondary to neoplasia, liver disease, or other systemic derangement). He got 1/2 unit total of packed red blood cells during and after surgery.

In surgery, wide lateral margins of the mass were obtained but we were unable to remove the fascial plane deep to the mass due to its location over midline. With very little subcutaneous fat. and the location of the mass over dorsal midline, it was impossible to dissect deeper tissue. The incision is long, extending from mid-scapula region to approximately the level of L3-4, but closed nicely with little tension. There was a fair amount of dead space, therefore 2 closed suction grenade drains were placed and managed for 2 days post-operatively with minimal production. Surgery, anesthesia, and recovery were uneventful. Pain was controlled with fentanyl CRI initially, and then transitioned to oral tramadol. IV antibiotics consisted of unasyn. Biopsy results are pending, and will be back by the end of next week.

On 3/3/13 [he] was on a visit with his owners, and due to his speedy recovery, was feeling quite bright and over-exerted himself on a walk outside. The walk was brief, but when he returned he was hypersalivating, retching, and had become markedly gas distended in his abdomen. A lateral abdominal radiograph confirmed gastric dilatation with early pyloric rotation (i.e.. early GDV). He was briefly anesthetized and intubated so an orogastric tube could be passed for gastric decompression. A large volume of air was removed, and a small amount of liquified ingesta. Immediately he was markedly less distended, and re-check radiograph confirmed correct placement of the stomach and pylorus. He was monitored the rest of the day in the ICU, and aggressive GI prophylaxis was commenced (Cerenia, Reglan CRI, famotidine, and small/frequent meals to promote GI motility). He did well the rest of the day, and the owners were counseled on the seriousness of GDV and the signs to look for at home. They know this would be a surgical, life threatening emergency.

[He] is a great, resilient dog, with very loving and committed owners. Please call if you would like to discuss this case in further detail. Please see plan for home care and medications below.

PATIENT CARE INSTRUCTIONS

DIET: please feed small, frequent meals in order to keep the GI tract moving. Only feed about 1/4 cup of food at a time. Do not feed from elevated food bowls, as this increases risk of GDV, and also avoid high fat foods (cheese, too many treats). Pill pockets are ok for medications but should not be used as treats when not giving the pills, unless a very tiny piece is given.

INCISION CARE: monitor daily for heat, swelling, discharge, malodor, or pus. These are signs of incision infection and should prompt immediate re-check. A tiny amount of blood-tinged fluid is normal for the next few days. He should allow gentle palpation of the incision daily, and if he acts like it hurts more than it did the day prior, please call or come in anytime. Sutures will need to be removed in 10-14 days. Please remove the lidocaine patches on either side of the incision tomorrow by gently peeling them off the skin. Dispose of them in a paper towel in the trash. DO NOT ALLOW LICKING (I know this may be difficult), but he must wear an E-collar all times unattended and you can try a t-shirt as well.

MEDICATIONS AND TREATMENTS

PAIN MEDICATIONS:

Tramadol 50mg: this is an opioid-derivative, side effects may include sedation. He may not need the full amount of medication, so after about 2 days please use your judgement based on his level of comfort.

Gabapentin 100mg: this is a pain medication that also has some anti-anxiety effects. Give every dose to promote him being quiet and resting so he can heal.

ANTIBIOTICS:

Clavamox 250mg: this is for the infection from the ulcerative mass, side effects of administration include GI upset, so if any vomiting or inappetence occur please call.

GI PROTECTION:

Famotidine (Pepcid) 10mg: this is a GI ant-acid medication. [He] has some changes in his blood work that could indicate chronic, low-grade, GI bleeding, and therefore should be on Pepcid daily. He will likely need this medication long-term, so please pick-up over the counter at any local pharmacy, as this is most cost-effective option. At the time of re-check exam/blood work we can decide if he needs to continue on this medication.

ITEMIZED MEDICATIONS

* gabapentin 100mg capsules 0 6 capsules administer 1 cap by mouth every 12 hours until gone Refills: None Next Dose: before bed Status: Dispense

* famotidine 10mg tablets 0 0 administer 1.5 tabs by mouth once daily until otherwise instructed by veterinarian Refills: None Next Dose: Status: over the counter

* CLAVAMOX 250mg tablets 0 14 tablets administer 1 tab by mouth every 12 hours until gone Refills: None Next Dose: with dinner Status: Dispense

* pill pockets for dogs pkg/30 0 1 bag Refills: None Next Dose: Status: Dispense

* tramadol 50mg tablets 0 10 tablets administer 1 tab by mouth every 12 hours for pain relief. Refills: None Next Dose: before bed Status: Dispense

FOLLOW UP INSTRUCTIONS

Call [your veterinarian] with an update next week and for biopsy results (call on Wednesday or Thursday for these, though it may take until Friday to get them finalized). Schedule a re-check/suture removal in 10-14 days, or with (ICU resident) schedules permitting. Call or return to the ER anytime if problems arise at home.

**Soap Text Created By - Technician: Surgery Report, FIRST - Updated on: 3/1/2013 3:18:01 PM**

DATES

Procedure/Study: 2013-03-01T15:18:03

DIAGNOSES

&amp;lt;Diagnosis&amp;gt;Ulcerated mass removal &amp;lt;/Diagnosis&amp;gt;

SURGICAL REPORT

The patient was placed in sternal recumbency, clipped and aseptically prepared. Using a #10 blade a circumferential incision was made around the ulcerated mass over the dorsum with approximately 1cm margins, for an approximate length of 20cm. Due to the location and thin body condition of patient, unable to get 1 fascial plane deep margin. The mass was dissected using sharp and blunt dissection. Bleeding was controlled with electrocautery. The mass was inked and submitted for histopathology. Two Jackson Pratt drains were placed, one on each lateral side, and sutured with a purse-string/finger-trap pattern of 3-0 Nylon. The deep layer was closed with 0 PDS in a horizontal mattress pattern. The superficial layer was closed with 2-0 PDS in a simple interrupted pattern. A subcuticular pattern was placed with 3-0 maxon in a simple continuous pattern. The skin was closed with 3-0 Surgipro in a cruciate pattern. The patient recovered from anesthesia uneventfully.

**Soap Text Created By - Technician: RAD Report, FIRST - Updated on: 3/3/2013 12:00:00 AM**

DATES

Date of Study: 2013-03-03T00:00:00

CASE ABSTRACT

Two days post-op mass removal over dorsum. Got worked up on walk with owner and started retching.

EXAMINATION DESIRED

Abdomen (R Lateral [GDV view])

REQUEST SPECIFICS

Outpatient / Inpatient

Location: Ward Stall

Tranquilizer (Y/N):

Anesthesia (Y/N):

RDVM Rads (Y/N):

Weight:

Drug/Isotope: Dose: Time:

IMAGING FINDINGS

ABDOMEN (1 view, 11:06:37) The cranial abdomen is not included and the projection is severely rotated:

The stomach is severely distended with gas and the pylorus is in an atypical location dorsal to the fundus and cranially angled. The pylorus is at the level of the mid-body, suggesting torsion. The small intestine are diffusely gas filled and normal in size. Incidentally, moderate spondylosis deformans is present at L2-3 and L7-S1.

ABDOMEN (1 view, 11:29:54): The cranial and caudal abdomen are not included in this study.

The stomach is in a normal position and contains a large volume of granular soft tissue material. The spleen is in a normal position and normal size. The small intestine are gas and fluid filled. Peritoneal detail is normal.

IMAGING CONCLUSIONS

Gastric dilation and volvulus with resolution of volvulus. This is compatible with the clinical history of gastric intubation.

**Soap Text Created By - Technician: Case Summary, FIRST - Updated on: 10/28/2013 6:44:12**

Service Date: 10/28/2013

DATES

: 2013-10-28T06:44:29

Admission: 2013-10-28T06:44:12

TO THE REFERRING VETERINARIAN

Dear Colleagues,

PROFESSIONAL REPORT

[He], a 17 yr old CM mix breed, presented to the our institution for euthanasia. On presentation he was recumbent and very weak and owners reported pain at home. Owners declined an exam. They elected to euthanize him and be present. 4mls of euthasol was injected and his passing was peaceful. No heart beat was auscultated following injection. They elected private cremation with a clay paw.

I am so sorry for the loss of your patient. Please let me know if you have any questions.

**CLE Case 2 biopsy report notes:**

Microscopic Findings:

Punch biopsy 1: Non-haired skin, nasal lesion

There is a thick lichenoid band of mixed inflammatory cells composed of lymphocytes, plasma cells, histiocytes, and smaller numbers of scattered neutrophils primarily in the superficial dermis. In some areas the inflammation obliterates the basement membrane and transitions to an interface dermatitis. There is disorganization of the basal cells, mild multifocal vacuolar change of the basal cells, and very rare apoptotic basal cells. Two neutrophilic intracorneal pustules (1 superficial and 1 deep) are present, but neither contains acantholytic cells. In the deeper dermis, there is also multifocal lymphoplasmacytic adenitis, and the inflammatory cells in there do not affect the glandular epithelium. There is also dermal fibrosis, marked acanthosis, and orthokeratotic hyperkeratosis. A few pigmented-laden macrophages are also present in the dermis.

Comment:

The lichenoid interface dermatitis is consistent with immune-mediated disease but the pattern of inflammation is not cleanly classifiable. Based on the microscopic features in these sections, the following differentials are favored: lupus (rare apoptotic basal cells), pemphigus foliaceous (neutrophilic intracorneal pustules), and pemphigus erythematosus (which shows features of both diseases). Pemphigus foliaceous was listed as a previous diagnosis from 5-6 years ago. Here, in these sections acantholytic cells are not present and either are eosinophils. A lack of these cells does not completely exclude a diagnosis of pemphigus foliaceous, as there is variability in the disease expression. It is possible that the chronicity of disease and treatments may have altered the cellular immune responses or dominant antigens, which ultimately could alter the morphology. Additional diagnostics may be necessary to rule in or out specific immune-mediated diseases. There is no evidence of mycosis fungoides.

**CLE Case 3**

**Soap Text Created By - Technician: Case Summary, FIRST - Updated on: 2/21/2014 1:15:03 PM**

Service Date: 02/21/2014

DATES

Discharge: 2014-02-22T18:37:37

Admission: 2014-02-21T13:15:05

TO THE REFERRING VETERINARIAN

Dear Colleagues,

DIAGNOSES

Provisional diagnoses:

No evidence of:

Final diagnoses: hypoadrenocorticism (Addison's disease), presented with an early crisis, pyodermatitis with mucocutaneous &amp; pedal lesions

Recommendations: see below

Procedures: blood work (blood gas analyses, complete blood count, chemistry profile), dermatology consult, skin biopsies

Outcomes: improved with IV fluids, steroids, and supportive care

Differential diagnoses: for skin rule outs include primary bacterial infection vs immune-mediated dermatitis with secondary bacterial infection vs neoplasia/cancer (unlikely)

CASE PROGRESS NOTES (ICU TRANSFER SHEET)

Time and Date Admitted: 02/21/2014 10:30 AM

Transfer Date:

History and Physical Exam Summary:

[He] is a 5 yr Castrated Male miniature pinscher who is a known Addisonian. [He] was diagnosed about two years ago. This morning, [he] had a low energy level and did not want to eat his food for breakfast, only treats (peanut butter, banana)- unusual for him. During an Addisonian crisis, [he] had presented before with inappetence, therefore his owner brought him to the ER. [He] is due for DOCP injection in four days, and he usually gets this every 32 days. Yesterday [he] had a normal energy level and appetite. At least three months ago, [he] developed lesions on the skin around his mouth. These have progressively been getting worse and the owner says he often scratches at them and at his eyes. Four days ago, the owners tried to wash the mouth area, soaking it with water to soften the crusts. The owner thinks this stressful event may have contributed to [his] state today.

[He] takes 1.25 mg pred (0.25 mg/kg) every third day normally, and DOCP 10.3 mg every 32 days. [He] received an extra dose of pred before his mouth cleaning attempt this week. This morning, [he] received 2.5 mg prednisone (0.5 mg/kg) before presentation to the ER. There is no history of coughing, sneezing, vomiting or diarrhea. About 6-7 months ago, [he] had ongoing colitis that eventually was managed with a diet change (now eating Sci Diet adult sm breed light dry). Since changing to the new diet, [his] owner thinks he has had increasingly hard, dry stool, and has been constipated and straining to defecate. She thinks this has gotten progressively worse over the 6-7 months. [He] is NOT up to date on his vaccinations - he has not been vaccinated since being diagnosed with Addison's disease.

MAJOR CAUTION. TRIES TO BITE. VERY WISE TO ALL MUZZLING/E COLLAR TRICKS.

S: BAR, trying to bite when the face is approached; mucous membranes not examined.

O:

T: not examined P: 160 R: shaking weight: 4.76 kg (previous weight- did not weigh today)

EENT:

PLN:

Cardio/Resp:

Abd:

UG: urinated in the lobby.

MS: ambulatory x 4

Integ: severe crusting all along commissures of the mouth; dark crusts; crusting around eyes.

Neuro: mentally appropriate, full exam not performed.

A1: hypoadrenocorticism

A2: decreased appetite r/o crisis due to A1

A3: decreased energy level r/o crisis due to A1

A4: perioral, periocular lesions r/o immune-mediated vs infectious vs allergic vs metabolic

A5: hard stool, straining to defecate r/o constipation/low fiber in diet.

P1: IVC, NOVA

P2: admit

P3: 1-2 mg/kg pred equivalent (dex sp)

P4: DOCP 10.3 mg IM once

P5: recheck NOVA

P6: Derm consult/ cytology or biopsy of lesions

P7: famotidine 1 mg/kg q12 hrs

P8: buprenorphine 0.10 mg q8 hrs

P9: feed Sci Diet Adult light

P10: IV LRS @ 130 ml/kg/day

Patient Condition:

stable

Initial and Current Therapy:

see above

Diagnostic tests completed and pending:

1. NOVA pH 7.286, PCV 53, TS 8.4, Na+ 139, K+ 5.19, Na/K 26.8, Lac 5.7, Gluc 121

Additional Requests Submitted:

none

Preliminary Diagnostic Results:

see above

Prognosis Given Owner:

good given we can get [him] through this early stage crisis. Derm lesions need workup, potentially derm consult, and biopsies. we will need sedation for this so waiting to get him thru the crisis first.

Client and Referring Veterinarian Communication Status:

Special Client Contact Information:

PROFESSIONAL REPORT

Presented for acute lethargy. Recurrent ulcerative/crusted skin lesions x 3-4mo, no specific work-up or treatment. They come and go but seem to clear up faster with increases in pred dose. Currently on ~0.3mg/kg Q3 days as a maintenance dose and monthly DOCP injections. Bloodwork (CBC/Chem) from the last visit last month was unremarkable. The skin lesions are focused around the mucocutaneous junctions of the mouth, periocular, and plantar surface of pes behind paw pad (hind limbs & forelimbs. He was hospitalized overnight and responded well to IV fluids, dexSP injections, and received his DOCP injection (4 days earlier than he was due for it anyway). Please see the full summary below in the client report.

Diagnostics:

2/21:

Blood gas analysis (admit): pH 7.286, PCV 53, TS 8.4, Na+ 139, K+ 5.19, Na/K 26.8, Azo 15-26 (BUN/Creat not working on NOVA), Lac 5.7, Gluc 121

Blood gas analysis (re-check): PH normalized, Na: K improved (29.9), Lac improved (3.0)

2/22:

Dermatology consultation with skin biopsies (mucocutaneous oral lesions &amp; pedal lesions sampled)

CBC: inflammatory leukogram

Chemistry: ALP mildly elevated (improved from previous chemistry 1/14)

*this blood sample was obtained during deep sedation with propofol and the mild anemia is likely a reflection of anesthetic change

Plan: see short course of higher prednisone taper dose below, and further plan to be made pending skin biopsies.

TO OUR CLIENT

CLIENT REPORT

[He] came to see us in the ER due to inappetence yesterday morning. He was due for his DOCP this coming Tuesday and had just seen [veterinarian] last month for his injection and routine blood work. He has been doing well, and blood work looked good. You report some skin lesions over the past few months, which have recurred recently and likely contributed to an early Addisonian crisis. When he came in he was dehydrated and had electrolyte changes indicative of Addison’s disease, but you caught his signs very early and the changes were mild. We started IV fluids and gave an injection of steroid at a higher dose than he normally gets along with his DOCP dose. He did very well thereafter and ate for us overnight (preferred wet food as his mouth was a bit sore).

Today we performed a consult with our dermatologist and took skin biopsies of the crusting along his mouth and from the spot behind his paw pad from one of his hind limbs. There was one absorbable suture placed at each site. There is a bacterial infection present, we just do not know yet if this is primary or secondary to an underlying skin disease, such as an immune-mediated process. The biopsies will help answer this and will be back next week.

We are sending [him] home with pain medication, antibiotics, and a liquid suspension to apply topically to affected skin (if he allows) to help soothe any raw/exposed skin. See below for medication plan details.

Thank you for bringing him in, it is always a pleasure to look after him!

PATIENT CARE INSTRUCTIONS

Feed soft food for the next few days until his mouth is feeling better.

Monitor as usual for signs of inappetence, lethargy, GI upset, or anything out of the ordinary that may suggest Addisonian crisis again.

Monitor biopsy sites daily for any excessive swelling, pain, or increased pus or discharge. No suture removal will be needed.

MEDICATIONS AND TREATMENTS

Prednisone: INCREASE dose to 5mg/day for the next 3 days, then 2.5mg for 3 more days, then back down to 1.25mg daily for 1 week, then we will decide where to go from there based upon how he is doing.

Simplicef (cefpodoxime): antibiotic for the skin, give with food as GI upset can occur on empty stomach

Omeprazole: antacid medication to protect GI tract while on higher dose of steroids, give for 10 days, then use in conjunction with future increases in prednisone doses when needed.

Pain medications:

Tramadol: give 2-3 times daily for pain relief for the next 3-5 days, then use your judgment based on his comfort. Side effects include sedation, so you can lower the dose if he is too sleepy or skip a dose if needed. Please use your judgment.

Miracle mouth wash: not meant to be ingested, but will not cause harm. Rare side effects include incoordination, excessive sedation, or neurologic deficits (i.e.. seizures). Stop administration and call us if concerned.

ITEMIZED MEDICATIONS

* MIRACLE MOUTH WASH ( per ml) 0 50 mLs administer approximately 1mL along mouth 3-4 times daily for pain relief Refills: 2 Next Dose: tonight Status: Dispense

* omeprazole 10mg capsules 0 30 capsules administer 1 cap by mouth once daily Refills: 2 Next Dose: tomorrow Status: Dispense

* cefpodoxime 100mg SIMPLICEF 0 14 tablets administer 1/2 tab by mouth once daily until gone or otherwise instructed by veterinarian Refills: None Next Dose: tonight before bed Status: Dispense

* tramadol 10mg/ml suspension 0 90 mLs administer 1mL by mouth up to 3 times daily as needed for pain relief Refills: 1 Next Dose: before bed tonight Status: Dispense

FOLLOW UP INSTRUCTIONS

Call next week (Wed. or Thurs) to discuss biopsy results.

**Soap Text Created By - Technician: US Report, FIRST - Updated on: 2/28/2014 9:21:49 AM**

Service Date: 02/28/2014

DATES

Date of Study: 2014-02-28T09:21:50

CASE ABSTRACT

Possible hepatocutaneous syndrome based on skin bx (skin lesions x 3-4mos). Hx of hypoadrenocorticism (Addison's) on pred and DOCP. Mildly elevated ALP.

EXAMINATION DESIRED

Ultrasound Abdomen

IMAGING FINDINGS

Some small hyperechoic urinary bladder luminal foci are suggestive of small bladder calculi.

The kidneys have prominent arcuate artery walls.

The gastrointestinal tract is within normal limits.

The liver and gallbladder are within normal limits. No splenic abnormalities are detected.

The adrenals remain small and difficult to visualize, being 2-4mm in thickness.

IMAGING CONCLUSIONS

Small adrenals consistent with the clinically reported hypoadrenocorticism.

Small cystic calculi. A urinalysis can be considered.

No evidence of hepatic disease.

**Soap Text Created By - Technician: Case Summary, FIRST - Updated on: 2/28/2014 1:14:17 PM**

Service Date: 02/28/2014

DATES

Appointment: 2014-02-28T13:14:18

Discharge: 2014-02-28T13:14:18

TO THE REFERRING VETERINARIAN

Dear Colleagues

DIAGNOSES

Adverse events :

Procedures: abdominal ultrasound, i-stat electrolyte panel, amino acid panel

Recommendations: continue antibiotics

Differential diagnoses: for skin lesions consider immune-mediated vs primary pyoderma/infection vs amino-acid deficient hepatocutaneous syndrome (less likely)

No evidence of :

Final diagnoses: Addison’s disease (Controlled), lymphocytic/histiocytic pyoderma (improving), mild liver enzyme elevation

Provisional diagnoses :

Outcomes :

PROFESSIONAL REPORT

Please see report below regarding abdominal ultrasound to rule out hepatocutaneous syndrome due to recent dermatology biopsies. [He] is improved on his antibiotics and higher dose of prednisone taper.

CLIENT REPORT

[He] came in for his ultrasound today. There were no abnormalities seen, the only finding we will keep track of is a small amount of debris within the bladder that may be early stone formation. These are incidental and are not causing him any problems. The liver looks normal, so the skin/liver disease (hepatocutaneous syndrome) is very unlikely at this point. I will reconvene with the dermatologists and be in touch about when we should next re-check his skin. We sent out an amino acid panel which will be back in 1-2 weeks. Amino acid deficiency is associated with certain skin diseases, but no changes need to be made to his diet or medications until this panel returns. His electrolytes today are normal, indicating that Addison's is well controlled.

Please call in 1-2 weeks to discuss results of pending bloodwork.

MEDICATIONS AND TREATMENTS

Continue medications as previously prescribed. We can decide if he needs a longer course of antibiotics based on how he does once the current course is completed.

Continue prednisone taper as planned. Once he has been on 1.25mg daily for one week we can go to every other day as long as he is doing well.

**Soap Text Created By - Technician: Derm Case Summary, FIRST - Updated on: 4/23/2014 3:58:55 PM**

Service Date: 04/23/2014

DATES

Appointment: 2014-04-23T15:58:55

TO THE REFERRING VETERINARIAN

Dear colleague,

DIAGNOSES

Final diagnosis: history of mucocutaneous pyoderma, resolved; ischemic dermatopathy

Recommendations: **** Please see below for details.

Procedures: physical exam, acetate tape cytology

PROFESSIONAL REPORT

[He] was presented to the Dermatology service on April 23, 2014 for a recheck of his perioral and periocular skin lesions which were biopsied at the end of February. At that time, due to evidence of mucocutaneous pyoderma, [he] was started on an 8-week course of cefpodoxime. The lesions are now resolved. [He] is a known Addisonian, and right now he is receiving prednisone 1.25 mg orally every third day. [He] is due for his DOCP injection in about four days, which he receives routinely every 32 days.

At home, [he] is doing well overall, and his energy level and appetite are described by the owner as excellent. [He] does not scratch at his face anymore, but his owners report that he does sometimes bite at his elbows and hocks, where he has had waxing/waning scaling lesions since a few months after diagnosis with hypoadrenocorticism in December 2011.

Physical exam was limited due to [his] anxious nature and the concern with stressing him with handling. Visual examination revealed scaling and crusting on the pressure points of his elbows and hocks. He also had some skin loss/pallor in the central part of some footpads, as well as hair loss and impression of raggedness on the pinnal margins. An acetate tape impression of the hock lesion revealed no infection. Due to the clinical presentation, we are most suspicious of ischemic dermatopathy. We have prescribed pentoxifylline.

If you have any questions, feel free to contact us.

Best regards,

CLIENT REPORT

Thank you for bringing [him] at our institution. You brought [him] to the Dermatology service on April 23, 2014 to recheck the skin lesions around his mouth and eyes which were biopsied at the end of February. At that time, a bacterial infection was diagnosed in the skin around his mouth and eyes, and [he] was started on cefpodoxime. After starting the antibiotic, [his] lesions around his mouth and eyes improved markedly and today they have completely resolved. [He] is known to have Addison's disease, and right now he is receiving prednisone 1.25 mg orally every third day. [He] is due for his DOCP injection in about four days, which he receives routinely every 32 days.

We are so pleased that [he] is doing well at home, and that his energy level and appetite are so much better than they have been in a long time. We are also very pleased that the skin problems around his mouth and eyes have resolved. We did take a sticky tape impression of the sore on his leg, which we examined under the microscope. We did not find any evidence of infection, which is great. However, the scabbing on his elbows and hocks, as well as the lightening of the skin on some of his footpads and the slight loss of hair on the edges of his ears, makes us concerned that these areas of thin skin are not getting sufficient blood flow, so the skin cells are dying. This condition, called ischemic dermatopathy, is not uncommon in small dogs. We are prescribing a medication which we hope will help increase blood flow with the goal of resolving the remaining skin problems. You will be contacted by the compounding pharmacy to confirm shipping and billing details.

MEDICATIONS AND TREATMENTS

Pentoxifylline is a medication that reduces inflammation and makes red blood cells more flexible, so they can better get to all areas of the skin. Possible side effects are rare but include hyperactivity, inappetence, and, even more rarely, nausea or vomiting. Pentoxifylline does not have adverse effects when taken with either prednisone or DOCP. We will start [him] on a full dose and will taper him over time. We do not expect to see full effects for up to 3 months.

Please continue prednisone at 1.25 mg (1/4 tablet) every 3 days, and DOCP injections every 32 days, as previously prescribed.

ITEMIZED MEDICATIONS

* pentoxyfylline 400mg 0 0 drops Refills: None&amp;lt;/refill&amp;gt;&amp;lt;status&amp;gt;Outside Script

FOLLOW UP INSTRUCTIONS

Please make an appointment with the Dermatology department in 2-3 months to recheck [his] skin. This appointment can be made by calling the Dermatology liaison,

**Soap Text Created By - Technician: Technician, Default FHSA - Updated on: 5/7/2014 8:51:37 AM**

Service Date: 05/07/2014

DATES

Discharge: 2014-05-07T08:51:37

Rx Fill: 2014-05-07T08:51:37

DIAGNOSES

Adverse events :

Final diagnoses: open for diarrhea; hx Addison's disease, mucocutaneous pyoderma, and ischemic dermopathy

No evidence of :

Recommendations :

Provisional diagnoses :

Differential diagnoses :

Procedures :

Outcomes :

PATIENT CARE INSTRUCTIONS

MEDICATIONS AND TREATMENTS

Metronidazole is a medication often used to help resolve diarrhea. If there is no improvement in a day or two we should regroup.

ITEMIZED MEDICATIONS

* metronidazole 50mg/ml susp 50mg/mL 20 mLs Give 1mL by mouth twice daily. Give for 5-7 days. Refills: None Next Dose: Status: Dispense

**Soap Text Created By - Technician: Technician, Default FHSA - Updated on: 5/28/2014 4:01:34 PM**

Service Date: 05/28/2014

DATES

Rx Fill: 2014-05-28T16:01:33

Discharge: 2014-05-28T16:01:33

DIAGNOSES

Procedures :

Differential diagnoses :

No evidence of :

Outcomes :

Adverse events :

Final diagnoses: Addisonian, Vasculopathy

Recommendations :

Provisional diagnoses :

CLIENT REPORT

Thank you for bringing [him] in today. We were unable to trim [his] nails today because he was getting stressed. We will continue on Friday or Saturday.

[He] received his DOCP today.

Based on the changes to [his] skin around the lip, we are continuing the antibiotic that helped clear this in the past.

As we discussed, please consult with dermatology regarding the pentoxyfylline.

ITEMIZED MEDICATIONS

* cefpodoxime 100mg SIMPLICEF 100mg 14 tablets Give 1/2 tablet one daily for 28 days. Refills: None Next Dose: Status: Dispense

**Soap Text Created By - Veterinarian - Updated on: 7/21/2014 1:57:32 PM**

7/21/2014 1:57:36 PM EXAM, GENERAL

Sores are doing well, top of nose is improved, periocular area is improved, unable to give medications because of severe GI issues so d/c after 4 days, chews occasionally but greatly improved, finished with antibiotics

currently on pred every third day (1/4 tablet), DOCP every 32 days, HW prevention every 30 days

no other concerns at this time

Subjective (S)

BAR, nervous, 4.45 kg

Objective (O)

Full exam not performed due to nervous nature/aggressive behavior

Small areas of alopecia and dryness on hocks and crusting around lips

Assessment (A)

A1: mucocutaneous pyoderma- improving

A2: ischemic dermatopathy- improving

A3: hypoadrenocorticism- well managed

**Soap Text Created By - Veterinarian - Updated on: 8/1/2014 2:36:41 PM**

8/1/2014 2:36:47 PM EXAM, GENERAL

Subjective (S)

[He] has been doing well at home. Owner has no concerns.

Objective (O) Unable to approach dog without being lunged at

BAR pink able to notice lip fold crusting - occurred in past, cleared with Simplicef - represcribed

Technician assisted administration of injection

Assessment (A)

A1:requires DOCP every 32 d 10.3mg IM

A2: lip fold crusting

Plan (P)

Inject DOCP carefully CAUTION

Notify owner to stop cefpodoxime 7 days after resolution of crusting

**Soap Text Created By - Veterinarian- Updated on: 9/23/2014 4:14:19 PM**

09/23/2014

History: Addisonian, being treated. Also being treated by derm.

Presenting complaint: 3-week history of intermittent diarrhea being treated with metronidazole and probiotics. Vomited today, once, yellowish fluid. Presented to ER for concerns over repeat of dehydration-induced Addisonian crisis.

9/23/2014 5:59:11 PM EXAM, GENERAL

Subjective (S)

BAR, nervous, caution. BCS 5/9

Objective (O)

T: not done due to temperament HR: 150 RR: 24 BW: 3.6kg

EENT: clear OU, no ocular discharge. clean AU. no nasal discharge. mild dental calculus.

PLN: no PLA palpated

H/L: normal sinus rhythm, no murmurs auscultated, strong synchronous pulses. normal bronchovesicular sounds.

Abd: non-painful, no masses

UG: externally normal neutered male

MSI: adequate muscle condition, clean intact haircoat, no sign of ectoparasites.

Assessment (A)

A1: Vomiting: r/o non-GI (Addison’s vs pancreatitis vs Cushing’s disease vs DM vs neoplasia) vs GI (infectious vs dietary indiscretion vs foreign body)

A2: Addison’s disease

A3: Chronic diarrhea: r/o non-GI (Addison’s vs neoplasia) vs GI (inflammatory vs infectious vs neoplasia)

Diagnostics: CBC/Chem WNL - identifying no chem changes (electrolytes normal), CBC ok - mild basophilia.

Plan (P)

1. IV fluid therapy 90ml/kg/day overnight

2. Cerenia

3. Famotidine

4. If continuing to vomit: AUS tomorrow

5. 4DX per owner request

Client comm:

Proactive measures tonight since he has gone downhill fast in the past when has had GI signs. Has had diarrhea for a few weeks and now vomiting. Discussed reasonable to check US again tomorrow since he could have a problem that is unrelated to Addison’s. Did not feel he needed urgent imaging on overnight but discussed that if he changes overnight that we would update and change plan. Owners great and ok with plan.

Estimate: 1500.

Prescribed - Dexamethasone SP 4mg/ml - FHSA (0.2)

Instructions - 0.05ml (0.2mg) IV once

4kg - Expires: 9/23/2015 No Refills

Prescribed - Cerenia 10mg/ml Maropitant (10)

Instructions - 4mg (0.4ml) IV once

4kg - Expires: 9/23/2015 No Refills

Prescribed - Famotidine 10mg/ml Injectable (8)

Instructions - 4mg (0.4ml) IV q12h - Expires: 9/23/2015 No Refills

9/24/2014 12:04:25 PM

Prescribed - Prednisone 5mg - FHSA (6)

Instructions - Please give 1 tablet by mouth once daily for the next 3 days, then give 1/2 tablet by mouth for 3 more days, then down to 1/4 tablet by mouth once daily for 1 week. - Expires: 9/24/2015 No Refills

**Soap Text Created By - Veterinarian- Updated on: 9/24/2014 11:34:16 AM**

Subjective (S): Comfortable overnight; no vomiting or diarrhea noted.

Objective (O)

T: not done due to temperament BW: 3.6kg

EENT: clear OU, no ocular discharge. clean AU. no nasal discharge. mild dental calculus. MM pink. normal cervical examination.

PLN: no PLA palpated

CV: normal sinus rhythm, no murmurs auscultated, strong synchronous pulses.

Resp: normal bronchovesicular sounds. eupneic

Abd: non-painful, no masses

UG: externally normal neutered male

MSk: ambulatory x4. BCS 6/9

Neuro: BAR n mentation.

Assessment (A)

A1: Vomiting: r/o non-GI (Addison’s vs pancreatitis vs Cushing’s disease vs DM vs neoplasia) vs GI (infectious vs dietary indiscretion vs foreign body) [ resolved ]

A2: Historic Addisons disease

A3: Chronic diarrhea: r/o non-GI (Addison’s vs neoplasia) vs GI (inflammatory vs infectious vs neoplasia)

Diagnostics: CBC/Chem WNL - identifying no chem changes (electrolytes normal), CBC ok - mild basophilia.

No evidence of low/lack of glucocorticoid (normal cholesterol, Alb, and GLU)

Plan (P)

Discontinue IVF, offer food, and send home for continued monitoring with short taper of pred

Client comm:

Proactive measures tonight since he has gone downhill fast in the past when has had GI signs. Has had diarrhea for a few weeks and now vomiting. Discussed reasonable to check US again tomorrow since he could have a problem that is unrelated to Addison’s. Did not feel he needed urgent imaging on overnight but discussed that if he changes overnight that we would update and change plan. Owners great and ok with plan.

**Soap Text Created By - Veterinarian- Updated on: 12/22/2014 7:14:07 AM**

12/22/2014 7:27:03 AM NEW VISIT (ER)

Presenting complaint: two-day history of inappetence and lethargy. Yesterday morning wouldn't eat his breakfast or take treats, extremely out of character. He did eat last night and this morning but slowly and without his usual vigor. Had one small bowel movement yesterday, small and firm and he has been very gassy. Had one episode of vomiting yesterday, bile, mucus, and food. Has been very lethargic at home. O says he gets into things often, particularly underwear.

Past pertinent medical history: Diagnosed with Addison's in 12/2011, managed here.

Medications currently administered at home: DOCP injection q 32 d. Pred 1.25 mg PO for two days, then off for one day. Gets Prilosec with pred. HW med this morning.

Dietary history: Science diet

Type of food: wet and dry

Amount per feeding:

Feedings per day: two

Visit is a referral: No

Bloodwork completed prior to arrival: No

Exam: CAUTION

Subjective (S): BAR, unable to assess mm

BCS: 6/9

Hydration: adequate

Objective (O)

T: NP; P: 132; R: 42; Wt: 5.2 kg

EENT: mild serous ocular discharge, no nasal discharge, ears clean, unable to assess dentition

PLN: wnl

H/L: NMA, NSR, normal BV sounds bilaterally

Abd: soft, non-painful, no organomegaly or masses appreciated

UG: externally normal CM

MSI: amb x 4, healthy hair coat, no ectoparasites noted

Neuro: mentally appropriate

Rectal: NP due to temperament

Assessment (A)

A1: inappetence, lethargy, vomiting - r/o gastroenteritis vs foreign body vs inadequate Addisonian control vs other

Plan (P)

P1: Abdominal radiographs - no obstruction evident

P2: NOVA, PCV/TS - K+ 3.64, Lac 2.5, PCV 50, TS 7.0

P3: LRS 100 mL SQ

P4: famotidine 1/4 tablet BID

Home for supportive care and monitoring

TS (FHSA): 7

PCV **: 50

**Soap Text Created By - Veterinarian - Updated on: 5/18/2015 2:22:53 PM**

Doing well at home. Gave 10.2mg (0.412ml) DOCP IM q30 days. iStat, 4DX.

Refilled Pred Rx: 1/4 (5mg tab) PO q2-7 days as needed

5/18/2015 2:42:40 PM

Prescribed - Prednisone 5mg - FHSA (80)

Instructions - Give 1/4 tablet by mouth every 2-7 days as needed. GIVE WITH FOOD - Expires: 5/17/2016 No Refills

5/18/2015 2:47:52 PM: TS (FHSA) 7.8

5/18/2015 2:47:52 PM: PCV ** 60

5/18/2015 2:48:06 PM: AGAP (i-STAT) 26

5/18/2015 2:48:06 PM: Hb 19.7

5/18/2015 2:48:06 PM: HCT 58

5/18/2015 2:48:06 PM: Creat 0.8

5/18/2015 2:48:06 PM: BUN 15

5/18/2015 2:48:06 PM: Glucose (i-STAT) 127

5/18/2015 2:48:06 PM: TCO2 15

5/18/2015 2:48:06 PM: iCa 1.33

5/18/2015 2:48:06 PM: Cl- 111

5/18/2015 2:48:06 PM: K+ 4.3

5/18/2015 2:48:06 PM: Na * 147

Lactate 5.6

Modified Soap Entry

6/23/2015 4:55:51 PM

Prescribed - DOCP 25mg/ml Percorten (10.2)

Instructions - Give 10.2mg DOCP IM every 32 days - Expires: 6/22/2016 No Refills

**Soap Text Created By - Veterinarian- Updated on: 7/28/2015 4:11:19 PM**

Doing well at home. Temperament seems better than last year as well.

On pred 2days on 1 day off (1/4 5mg)

Brief examination prior to injection.

H/L: wnl

ABD: wnl

MSI: some crusting around commissures of mouth and lateral canthus of the right eye (some squinting present)

A: Addisonian

A: Previous diagnosis of vasculitis

A: Recurrent antibiotic responsive dermatitis

P: DOCP 10.2mg IM q32 days

Simplecef 50mg PO SID

7/28/2015 4:18:22 PM

Prescribed - Cefpodoxime 100mg Simplicef - FHSA (30)

Instructions - Give 1/2 tablet by mouth with food once daily for 10 days. Repeat course of this medication as needed until skin clears. - Expires: 7/27/2016 No Refills

**Soap Text Created By - Veterinarian - Updated on: 10/8/2015 11:52:00 AM**

10/8/2015 11:52:05 AM EXAM, GENERAL

Subjective (S)

[He] is a 6-year-old CM miniature pinscher that is presenting today because the owner wants to have the teeth cleaned and needs a course of therapy needed to allow him to get teeth treated. Tick bite on Thanksgiving day 2012 and went into an Addisonian crisis and went into a coma. This was when he was diagnosed with Addison's dz. High-stress environment stops eating and gets fluids and steroids, two episodes total since diagnosis of crises. Not on flea/tick medicine. His eyes, ears, pads gets really dry skin and crusts, sees derm for those issues. Chicken allergy. No V/D/C/S. Doesn't like to drink, water down his food. Urinating normally. Vaccine titers in 2013 and were normal, does not vaccinate. No seizures. Travel history just to cape. Only pet at home. Energy good and good appetite. Cleaning ears once a month. Scratches his ears. Owner notes weight gain. Acquired form a breeder when 14 weeks old.

Diet: science diet (can and dry): 1/2 can and 1/4 cup dry, BID.

Medications:

Prednisone 1.25 mg two days on and one day off

Cefpodoxime 5mg PO BID (stops on Sunday).

Prilosec 5mg every third two days on and one day off

DOCP injection: 10.5 mg every 32 days

Objective (O) ABBREVIATED EXAM DUE TO BEHAVIOR

Mentation: BAR, nippy BW: 5.7 kg

EENT: Bright eyes, mild lenticular sclerosis, crusting around the eyes. Crusting around the commissure of the mouth.

H/L: HR 120, NSR, NMA (Dr. Mahony)

MSI: Overall clean coat, crusting around eyes and commissure of the mouth.

Assessment (A)

A1: Hypoadrenoscorticism - controlled on DOCP injection and prednisone.

A2: Crusting around eyes and commissures of the mouth - seen by dermatology and started on cefpodoxime.

Plan (P)

P: Discussed recommendations for [his] dental.

Prescribed - Prednisone 1mg - FHSA (60)

Instructions - Give one tablet by mouth once daily for two days then try skipping the third day - Expires: 10/7/2016 6 Refills

**Soap Text Created By - Veterinarian- Updated on: 4/12/2016 2:24:00 PM**

Weight: 5.2 kgs

Presented for follow-up on mucocutaneous dermatitis. O reports ever since pet's Addison's diagnosis about 5 years ago he gets waxing and waning crusting lesions: around the mouth, ear tips, paw pads, hocks, and elbows

**Soap Text Created By - Veterinarian- Updated on: 1/1/2017 8:42:09 AM**

Here for DOCP injection.

Owner reports that [he] has had more crusting around the eyes. Slightly improved today. Owner says that [veterinarian] refilled pred. for p/u today. Also restarting antibiotic

Admin DOCP IM as prescribed.

(Tai)

**Soap Text Created By - Veterinarian- Updated on: 6/13/2017 2:41:16 PM**

Presenting for DOCP injection. 10.3mg given IM.

Otherwise doing well at home. Has some crusting/dermatitis around the eyes, elbows and hock.

**Soap Text Created By - Veterinarian- Updated on: 2/9/2018 2:34:46 PM**

Subjective -severe ulcerative lesions at MC junctions. Otherwise unchanged, but unfriendly (normal for Rocco)

Objective See above,. severe MC lesions-mostly lips and OS

Assessment R/O pemphigus etc.

Plan Immunosuppressives, then biopsy if needed.

LR

**Soap Text Created By - Veterinarian - Updated on: 2/22/2018 2:26:10 PM**

here for DOCP/

skin and face better.

LR

**Soap Text Created By - Veterinarian- Updated on: 2/27/2018 1:18:42 PM**

Hx: 5 to 6 days of tachypnea, did not eat this morning, Owner also concerned about distended abdomen. The suspected hypersensitivity reaction around the commissure of the lips is much improved compared the past couple weeks.

pphx: Addisonian, ischemic dermopathy, intermittent colitis

S: BAR, nervous (fractious); HR 150, RR 45-60 at rest

O:

EENT: not assessed

PLNS: mandibular not palpated

H/L: bilateral soft velcro; NSR (no murmur auscultated, but difficult to hear heart)

ABD: tense, not distended, nonpainful

U/G: externally normal

MSI: dorsal fat pads consistent with prednisone administration

A: increased respiratory rate and effort r/o pneumonia (infectious vs drug reaction vs other) vs interstitial disease vs cardiac (e.g., LCHF) vs other

A: owner concern about distended abdomen r/o dorsal fat pads/hepatomegaly vs aerophagia vs increased intraabdominal fat vs other

A: Elevated liver enzymes r/o secondary to respiratory disease vs hepatopathy (infectious, inflammatory, toxin, neoplastic) vs other

P:

Sedation (OBAG protocol)

CBC/Chemistry/Nova (UA if possible)

CXR/AXR

Addendum: CXR revealed diffuse interstitial changes and a prominent PA. Also, LE elevation is worse than 2 weeks ago.

A: Diffuse interstitial lung pattern r/o interstitial disease (e.g., interstitial pneumonia (toxo, others) vs IPF vs other)

P: CT wash on Friday; Rerun bloodwork and add 4dx +/- FNA/Biopsy

JB

**Soap Text Created By - Veterinarian- Updated on: 3/8/2018 2:54:01 PM**

Presented for recheck radiographs for interstitial pneumonia/disease diagnosed last week.

S: BAR

O:

Lungs: able to listen right lung field, less crackles compared with exam last week

Several small crusting lesions at the commissure of the lips bilaterally

A: interstitial lung disease r/o drug reaction vs other

A: hx Addisonian

A: hx ischemic dermopathy

P:

Met check: interstitial changes subjectively improved from last week

Recheck again in 1 month

Continue: High dose pred (5mg PO BID), Finish Baytril/SAMe

**Soap Text Created By - Veterinarian - Updated on: 8/1/2018 3:22:26 PM**

Percortin administered IM (10.4mg)

5mg pred per day; owner still notes some shortness of breath; some crusting around mouth and ears.

**Soap Text Created By - Veterinarian - Updated on: 8/21/2018 5:31:38 AM**

NEW VISIT (ER)- Seizure

Presenting complaint: Possible seizure episode over 12 hours after ingestion of

Referral visit?

Diagnostics completed prior to visit

HISTORY:

Signalment:

Current History: Yesterday (8/20/18) [he] ingested 300mg of Cardizem (approx 53m/kg dose) (Diltiazem) around 4pm. Tried to induce vomiting with hydrogen peroxide and did not vomit. Was fine at home. This morning around 4 am after he got his prednisone he had what the owner calls a seizure.

Legs were flailing and on his side and back, no urination or defecation, was responsive when he was flailing.

Prednisone 5mg: 1/2 tab twice a day

Neurologic History:

Date of the event? 8/20/18

Description of the event: On his back flailing his legs.

Responsive during the episode? Yes

Duration of the event? 7-8s

Association with excitement/exercise vs. relaxation vs nothing: After pill administration

Urination or defecation during event? No

Vomiting or ptyalism during event? No

Post seizure behaviors? Duration of post-ictal period? Breathing heavy and went to crate.

History of prior seizure-like episodes? None

Duration of time between events? N/a

What time did/do the events occur? N/a

When was the most recent event? Yesterday

On any current anti-epileptic drugs? none

Other current medications: Prednisone 2.5mg PO q12h, DOCP every 32 days.

Diet: Science diet dry and wet

EXAM:

S: Bright, alert, and responsive

O: Wt - 5.6kg P: 120bpm R: 60bpm

BCS(1-9): 5/9

MCS(normal,mild,moderate,severe): Mild

Hydration: Euhydrated

EENT: Clear corneas, Iris atrophy OU, clean ears AU

PLN: No peripheral lymphadenopathy

C/V: No murmur or arrhythmia, strong pulses

RESP: WNL

ABD: non-painful

GU: externally normal neutered male

MSI: ambulatory x4, crusting on lips bilaterally

NEURO: CN intact, mentally appropriate, no CP def.

Pain Present(YorN)? Pain Score(0-4): 0/4

RECTAL: NP

ASSESSMENT:

A1: Collapse: r/o neuro (vascular vs. neoplasia) vs. syncope

PLAN:

EKG

BP

NOVA/PCV/TS

Offer neurology consult and day monitoring

TFAST

Diagnostics completed:

NOVA: Lactate 5.9

PCV/TS: 47%, 7.0

BP: 100

EKG: Normal sinus rhythm

TFAST: No effusion, normal ratio

Diagnostics pending: None

Client communication: We discussed that we do not believe that [he] ingested the pills, the high dose of medication would have been enough to stop his heart or show more clinical signs. He did have an episode of collapse. Recommended that [he] stay for continued monitoring during the day and for a neuro consult. The owner elected to take him home to monitor as she will be home during the day. She will consider a neurology consul when she follows up with [veterinarian] for DOCP injection next week.

**Soap Text Created By - Veterinarian - Updated on: 9/28/2018 8:45:20 AM**

Presenting for DOCP injection 10.3mg IM q32 days

Continue cepodoxime (50mg PO SID) for skin lesions (seem worse at change of season)

JB

**Soap Text Created By - Veterinarian- Updated on: 11/20/2018 4:47:52 PM**

11/24/18

BAR, eating/normal stool

Some crusting around the commissure of his lips.

P: continue food/water/walks/prednisone

**Soap Text Created By - Veterinarian- Updated on: 2/1/2019 8:54:51 AM**

Technician appointment

10.3mg DOCP given IM (epaxial).

Significant lesions on feet, around mouth and eyes. Recommended starting simplicef and consulting with a dermatologist due to previous drug reaction to cyclosporine/pentoxyphylline.

**Soap Text Created By - Veterinarian- Updated on: 3/5/2019 9:11:14 AM**

DOCP given. Skin very bad. had cyclosporine ILD, now breathing fine.

**Soap Text Created By - Veterinarian- Updated on: 5/7/2019 7:38:44 AM**

Presented for DOCP injection.

Doing well at home. Less fractious since some changes at home.

Still struggling with derm issues but O feels improving and seems to do better with sunshine.

Reference to cyclosporin related interstitial lung disease in record, appears to be doing well.

Generally appears healthy with some skin lesions. Vitals normal.

P:
- ISTAT: Na+ 142, K+ 4.9

- DOCP 10.30 mg IM once

**Soap Text Created By - Veterinarian - Updated on: 9/2/2019 10:50:23 PM**

**Subjective**

NEW VISIT (ER)

Presenting complaint: Inappetence, blood in the stool

Referral visit? N, long term patient here

Diagnostics completed prior to visit: N/a

HISTORY:

Signalment: 11yo CM Min Pin

Current history:

3 weeks ago, p was started on Meloxicam liquid for his back pain and given his routine DOCP injection. Three days ago, the owner noted blood in the stool and that patient turned his nose up at food, and stopped this medication. He would not eat his dog food yesterday, but ate hamburger and rice. Today he wouldn't eat that, but would eat chicken. The o feels he is more lethargic today, and was panting a lot when he woke up. P has had no vomiting, but has possibly been gagging/hard swallowing. Had some diarrhea, but has resolved.

Prior medical history: Known Addisonian, managed through ECC faculty

Current medications: Last NSAID dose 3 days ago, 5mg Prednisone SID (last two days has gotten 7.5mg PO SID)

EXAM:

S: QAR

O: Wt - 6.9 T: 101.4 P: 152 R: 32

BCS(1-9): 5/9

MCS(normal,mild,moderate,severe): Mild generalized muscle wasting

Hydration: 7% dehydrated, mm pink, tacky, CRT 2s

EENT: Eyes clear, no discharge OU, no nasal discharge, ears clean non-inflamed

PLN: No PLA palpated

C/V: NMA, NSR, FPSS

RESP: Eupneic, no crackles or wheezes auscultated

ABD: Suspect cranial organomegaly, no fluid or masses palpated

GU: Externally normal CM

MSI: Ambulatory x4, mild generalized muscle wasting, crusting, and scabbing on elbows

NEURO: Full neuro exam NP, mentally appropriate

Pain Present(YorN)? N Pain Score(0-4): 0

RECTAL: NP

ASSESSMENT:

A1: Blood in stool r/o GI ulceration vs. Addison’s vs. other

A2: Hypotension r/o hypovolemia vs. other

PLAN:

10mL/kg LRS bolus then 60mL/kg/d

Pantoprazole 1mg/kg IV q12

Monitor BPs

CBC/Chem held

Diagnostics completed:

BP before fluids: 79/58

BP after fluids: 143/121

NOVA: Lac 6.5, Creat 1.9 (BUN unavailable)

PCV/TS: 62/8.4

Diagnostics pending:

N/a

9-3-19

Bar- feels more like [him], limited PE associated with this.

No changes in PE, eating well this am.

**Soap Text Created By - Veterinarian - Updated on: 9/9/2019 11:07:55 AM**

Patient presents for regularly scheduled DOCP injection. Has been doing well at home per owner, with the exception of a waxing and waning appetite (still eating treats/human food very well).

EXAM:

S: BAR

O: Wt - 6.3 T: 101.2 P: 140 R: 30

BCS(1-9): 5/9

MCS(normal,mild,moderate,severe): Mild generalized muscle wasting

Hydration: Euhydrated, MM pink/moist, CRT 2s

EENT: Eyes clear, no discharge OU, no nasal discharge, ears clean non-inflamed. Moderate to marked dental calculus and gingivitis.

PLN: No PLA palpated

C/V: NMA, NSR, FPSS

RESP: Eupneic, no crackles or wheezes auscultated

ABD: Suspect cranial organomegaly, no fluid or masses palpated

GU: Externally normal CM

MSI: Ambulatory x4, mild generalized muscle wasting, crusting, and scabbing on elbows and in axillary/inguinal regions.

NEURO: Full neuro exam NP, mentally appropriate, mild pelvic limb proprioceptive ataxia noted with scuffing.

Pain Present(YorN)? N Pain Score(0-4): 0

RECTAL: NP

Plan:

- DOCP injection (12mg IM)

- Send home refill of Metronidazole if diarrhea develops again

- Send home Ondansetron to determine if nausea is component of waxing / waning appetite.

David Conway, DVM

**Soap Text Created By - Veterinarian- Updated on: 11/13/2019 7:15:33 AM**

**NEW VISIT (ER):**

Presenting complaint: acutely down in hind

Referral visit? No

HISTORY:

Signalment: 11 yo MC Min Pin

Current history: Progressive hind end weakness. This morning, down in hind end and non-ambulatory. Vomited once this morning and inappetent.

Prior medical history: severe pulmonary hypertension, Addison's disease

Current medications: Prednisone, sildenafil

EXAM:

S: QAR

BCS(1-9): 4

MCS(normal,mild,moderate,severe): mild diffuse

Hydration: 5%

EENT: Eyes clear, no discharge OU, no nasal discharge, ears clean non-inflamed. Moderate to marked dental calculus and gingivitis.

PLN: No PLA palpated

C/V: NMA, NSR, FPSS

RESP: Eupneic, no crackles or wheezes auscultated

ABD: Suspect cranial organomegaly, no fluid or masses palpated

GU: Externally normal CM

MSI: non-ambulatory paraparesis, mild generalized muscle wasting, crusting and scabbing on elbows and in axillary/inguinal regions.

NEURO: Weak to absent menace OU, mentally appropriate, absent hind limb proprioception, voluntary motor intact

Pain Present(YorN)? N Pain Score(0-4): 0

RECTAL: NP

ASSESSMENT:

A1: Non-ambulatory paraparesis

A2: Pulmonary hypertension

A3: Hypoadrenocorticism

PLAN:

- Neuro consult

- Humane euthanasia

**CLE Case 3 biopsy report notes:**

Microscopic findings:

Multiple punch biopsies resulting in 9 haired skin sections are examined. There is a lichenoid band of marked inflammation of the dermoepidermal junction and superficial dermis with extension into the mid-dermis (at the level of the adnexa) also affecting the follicular epithelium and rarely the sebaceous glands. The inflammatory cells in these regions are predominantly lymphocytes, plasma cells and histiocytes. There is also superficial epidermal ulceration and marked serocellular and fibrinous crust formation with suppurative inflammation with numerous colonies of trapped cocci. There is also multifocal follicular keratosis, and superficial keratosis. There are many areas where the inflammation is interface and extends into the epithelium, associated with basilar epithelial cell vacuolar degeneration, hypertrophy and hyperplasia. There is also multifocal moderate to marked pigmentary incontinence.

Comment:

Helpful differential clinical diagnoses were listed on the request. Comments with regard to the microscopic examination of these sections follow each, listed below:

Mucocutaneous pemphigus – clefts are not a feature

Pemphigus foliaceous – some features but not lots of acantholytic cells

Mucocutaneous pyoderma – some features present

Hepatocutaneous syndrome – does not have the trilaminar appearance

Neoplasia – no evidence

Overall, microscopic examination shows features of mucocutaneous pyoderma and pemphigus foliaceous. However, there is marked inflammation associated with the bacteria. It is therefore, difficult to determine which responses are driven by bacterial factors and which responses are driven by host factors (i.e. immune mediated). The classic cellular features of the immune mediated diseases are not observed; however, the general pattern of a lichenoid interface dermatitis is strongly suggestive of an immune-mediated component.

**CLE Case 4**

**Soap Text Created By - Technician: Technician, Default FHSA - Updated on: 5/28/2014 7:16:47 PM**

Service Date: 05/28/2014

DATES

Discharge: 2014-05-28T19:16:49

Admission: 2014-05-28T19:16:49

TO THE REFERRING VETERINARIAN

DIAGNOSES

Adverse events :

Procedures: Ocular exam

Recommendations :

Outcomes :

No evidence of :

Final diagnoses: Dry eye concurrent with possible systemic skin disease

Provisional diagnoses :

Differential diagnoses :

CLIENT REPORT

Thank you for bringing [her] in today.

Upon presentation, [she] was bright and alert. She had severe mucopurulent (pus) discharge in both eyes, with heavy crusting and matting of hair around the eyes. There was inflammation and erosion of the tissue surrounding the eyelids. Both corneas were affected with fibrosis and neovascularization, most severe in the right eye. Both eyes appeared dry at examination. Her vision and pupillary responses appeared normal.

At this time we would recommend that [she] make an appointment with a dermatologist, possibly here at our institution. We believe that treatment of her systemic skin issue will actually allow her eyes (and other issues) to improve. If you do decide to make an appointment with the dermatology service here we would like to recheck her eyes at the time of your appointment, to make sure that nothing is progressing.

Thank you for bringing [her] in to see us today. She is a very sweet girl and we hope to get to the bottom of her ailment soon

MEDICATIONS AND TREATMENTS

Please contact Pharmacy to arrange for payment and delivery information for [her] Tacrolimus prescription.

Try to keep the area around [her] eyes clean. You can begin using warm tap water compress using a gentle cloth (makeup removal pads work well) and then carefully clean the area with a dry cloth.

Please continue with your current medications prescribed by your referring veterinarian. Once you receive the compounded Tacrolimus from Pharmacy, you can discontinue application of the Optimmune drops and use the cyclosporine instead. Try to apply the Refresh eyedrops

ITEMIZED MEDICATIONS

* Tacrolimus 0.03% 0 0 bottle 1 drop into each eye twice per day Refills: None Next Dose: Status: Outside Script

* Refresh eyedrops 0 0 bottle 1 drop into each eye three times per day Refills: None Next Dose: Status: Continue

* Optimmune ointment 0 0 tube 1/2 inch strip into each eye twice per day Refills: None Next Dose: Status: Continue

FOLLOW UP INSTRUCTIONS

If you will sche3dule an appointment with the dermatologist at our institution, we can recheck [her] eyes when she is here. Otherwise, we would suggest a recheck in 4 weeks. Please do not hesitate to call if you have any questions.

**Soap Text Created By - Technician: Technician, Default FHSA - Updated on: 5/30/2014 9:06:58 AM**

Service Date: 05/30/2014

DATES

Appointment: 2014-05-30T09:06:58

TO THE REFERRING VETERINARIAN

Dear Colleague,

DIAGNOSES

underlying atopic dermatitis and perineal fistula, KCS

ulcerative and depigmented skin lesions around mouth, eyes, nose-- biopsies pending; pending cbc/chem; pending deep tissue culture

CASE PROGRESS NOTES (ICU TRANSFER SHEET)

eye meds:

Refresh Celluvisc lubricant eye gel

optimmune

neopolybac

rinse with saline solution

since dec

wearing cone since dec

Chronic dry eye - optimmune, refresh

Metacam SID for pain

born with cranial mandibular osteopathy - on pain meds entire first year of life

problem with licking foot - cefpodoxime 1x/day for 20 d and gold bond (has helped with foot licking)

hydroxyzine for pruritus 2x/day - on all last summer, stopped over winter, started again approx 1 month ago

then saw that she started to limp and her paw was swollen

on hypoallergenic venison royal canin and hypoallergic dog treats, gets food scraps

stopped scratching lately

famotidine for stomach

SAMe 1x/day

anal fissures - protopic (tacrolimus) 0.1% started on July 2013 until end of Dec 2013 b/c got better; started on it again last weekend

hasn't been groomed since December

PROFESSIONAL REPORT

[She], a 7-year-old spayed female West Highland terrier, presented to our institution for evaluation of severe skin disease periocularly and periorally. Her owner reported that she is on a number of ophthalmologic treatments, including Optimmune, triple antibiotic ointment, and Refresh Celluvisc drops. She was also recently on a corticosteroid eye drop, which seemed to worsen her condition.

In addition to her eye medications, [she] is on hydroxyzine (chronically due to a history of pruritus of the paws, which is likely secondary to atopic dermatitis), cefpodoxime (for a recently diagnosed pododermatitis which has improved with the medication), SAM-e (after bloodwork in August showed some elevated liver enzymes), and topical tacrolimus (for a perineal fistula). She has also recently been prescribed Clavamox and Metacam. She has been on a Royal Canin limited ingredient diet, although her owner reports that she sometimes gets other food at home.

On presentation, we noticed severe ulceration and crusting around both eyes, with neovascularization across both corneas and severe mucopurulent discharge of both eyes. There is ulceration and crusting around the oral commissures. There is depigmentation immediately below the nose, around the eyes, and around the mouth. There is hyperpigmentation and scarring from a perineal fistula at 5:00, which is very minimally open. There is erythema and moist debris in between the paw pads on the left front foot.

There are many differential diagnoses for these lesions, including mucocutaneous pyoderma, autoimmune disease (Pemphigus erythematosus, discoid lupus erythematosus), and neoplasia. We performed a biopsy, deep tissue culture, and a CBC/chem. The CBC/chem showed that [her] liver enzymes are still elevated (ALP 846; ALT 93; AST 66; CK 1429; amylase 1682), however, this is slightly improved from her liver panel in August. We recommended that her owner continue with SAM-e and monitor her liver values regularly.

Due to the severity of the ulceration around her eyes, we elected to perform the biopsies around the mouth, as the tissue there shows similar lesions. We will base a treatment plan on the results of these diagnostics. In the meantime, our skin cytology have shown numerous cocci. We prescribed clindamycin out of concern that she is resistant to the cefpodoxime. The results of the deep culture may indicate that a different antibiotic should be used.

Thank you for partnering with us in [her] care. Please do not hesitate to contact us with any questions or concerns.

CLIENT REPORT

[She] was diagnosed with cranial mandibular osteopathy when she was a puppy and was on chronic pain medications when she was young. She has a long history of licking her paws. Hydroxyzine was prescribed to try to help her paws, but you are unsure whether this has helped. She is also on a limited ingredient diet, although you mentioned that she sometimes gets other treats. Some routine bloodwork last August showed elevated liver enzymes, so [she] has been getting a SAM-e supplement since then. She has also been getting Refresh Celluvisc lubricant eye gel after being diagnosed with keratoconjunctivitis (dry eye) last summer. Your vet believes that the skin around her eyes may have gotten worse after treatment with eye drops containing prednisone.

You reported that she had an ulcer in one eye last winter which was treated successfully. After a trip to the groomer this past December, she had some small black crusts on her eyes. After examining [her] eyes, your vet prescribed optimmune and a triple antibiotic ointment with prednisone which she has been on since then. Her eyes have been getting progressively worse and she has also required pain medication (Metacam) recently. Famotidine has also been used to help with some indigestion that she developed due to all of her medications.

You visited the our institution, who diagnosed her with KCS and recommended that you see us.

On physical examination, we noticed severe ulcerations and crusting around both eyes, with neovascularization across both corneas and severe mucopurulent discharge of both eyes. There is ulceration and crusting around the oral commissures. There is depigmentation immediately below the nose, around the eyes, and around the mouth. There is hyperpigmentation and scarring from a perineal fistula at 5:00, which is very minimally open. There is erythema and moist debris in between the paw pads on the left front foot.

There are many differential diagnoses for these lesions, including mucocutaneous pyoderma (deep skin infection), autoimmune disease (pemphigus erythematosus, discoid lupus erythematosus), and neoplasia. To figure out what is going on with [her], we have sedated her and performed a biopsy, deep tissue culture, and bloodwork. Due to the severity of the ulceration around her eyes, we elected to perform the biopsies around the mouth, where similar lesions were. We will get the results of these tests throughout the next week, and we will contact you with a treatment plan. In the meantime, our skin cytology have shown numerous cocci bacteria, so we are going to send home a different antibiotic, out of concern that she is resistant to the cefpodoxime. We may need to change the antibiotic pending the culture.

Thank you for entrusting us with [her] care. We will be contacting you as soon as we get the test results back. Please do not hesitate to contact us with any questions or concerns in the meantime.

Sincerely,

PATIENT CARE INSTRUCTIONS

1. Biopsy sites: please monitor for swelling, discharge, or any other concerning issues, and contact us.

2. The sutures will need to come out in 10-14 days.

MEDICATIONS AND TREATMENTS

1. Eye medications: continue these as prescribed.

2. Clindamycin: This is a different family of antibiotics than what she's seen, so she is less likely to be resistant to it. However, we may change this, pending the culture. Give with food and call if GI upset is noted.

3. Protopic- continue to apply this to the perineal fistula as directed.

ITEMIZED MEDICATIONS

* clindamycin 150mg capsules 0 20 capsules Give 1 capsule by mouth twice daily until culture results are back. Give with food. Refills: None&amp;lt;/refill&amp;gt;&amp;lt;status&amp;gt;Dispense

FOLLOW UP INSTRUCTIONS

We will contact you with test results as they come in and formulate a plan based on that.

**Soap Text Created By - Veterinarian- Updated on: 6/16/2014 9:02:27 PM**

**1**6/16/2014 9:02:42 PM NEW VISIT (ER)

Presenting complaint: vomiting, bloody diarrhea, and not eating since June 1

Past pertinent medical history: Vomiting, bloody diarrhea, and not eating for about 2 weeks. All starting the day after being put under anesthesia for skin biopsies around the mouth. Seems to be drinking a lot, History of bacterial infection with a possible immune-mediated component around mouth, nose, and eyes (diagnosed the end of May at our institution). Also has been diagnosed with KCS (end of May) at our institution.

Medications currently administered at home: Artificial tears OU, tacrolimus OU, Baytril SID, sucralfate, Cerenia, Pepcid, metronidazole BID, Protopic on anal fistula. Owner is unsure of doses. Currently not on heartworm preventative or flea/tick preventative.

Dietary history:

Type of food: Currently syringe feeding baby food

Visit is a referral: Yes

Bloodwork completed prior to arrival: Yes (CBC/chem/UA)

Neutrophilia (13482), lymphopenia (1049), azotemia (BUN=39, Crea=1.9), hypoproteinemia (5.4), hypoalbuminemia (1.7), acidotic, bacteriuria, increased ALP (572), increased CK (463), hyperphosphatemia (6.9), decreased bicarbonate (8)

UA: 1.022, blood +1, bacteria +2, protein +3, WBC 2-5/hpf, trace ketones

Referred for specific test: AUS

Exam:

Subjective (S): QAR, slightly painful, mm=pink, CRT < 2sec

Objective (O): Wt=8kg, Temp=101, HR=84, RR=28

EENT: mucopurulent ocular and nasal discharge bilaterally, conjunctival hyperemia, ears clean AU, Ulcerative and depigmented skin around mouth, eyes, and nose, mm=pink, CRT < 2sec

PLN: Enlarged prescapular LNs that seem painful when palpated, Submandibular and popliteal LNs WNL

H/L: HR=84, NSR, no murmurs auscultated, RR=28, normal BV sounds

Abd: Non-painful, no masses or organomegaly palpated

UG: Normal external appearance of spayed female, no discharge

MSI: Ambulatory x4, symmetric and normal musculature

Neuro: Normal mentation, CNs intact, palpebral and menace present OU

Rectal: perianal fistula that is not swollen or erythematous

Assessment (A)

A1: Vomiting - R/O Renal disease (PLN, pyelonephritis) vs dietary indiscretion vs GI ulceration vs drugs (antibiotics, NSAIDs) IBD vs inflammatory vs infectious vs neoplasia

A2: Diarrhea - R/O Renal disease (PLN, pyelonephritis) vs dietary indiscretion vs GI ulceration vs drugs (antibiotics, NSAIDs) IBD vs inflammatory vs infectious vs neoplasia

A3: Anorexia - R/O Renal disease (PLN, pyelonephritis) vs dietary indiscretion vs GI ulceration vs drugs (antibiotics, NSAIDs) IBD vs inflammatory vs infectious vs neoplasia

A4: UTI from RDVM

A5: Azotemia - R/O prerenal, renal (PLN, pyelonephritis)

A6: Ulcerative and depigmented skin around mouth, eyes, and nose - diagnosed with severe bacterial infection with an immune-mediated component

A7: Perianal fistula

Plan (P)

Recommended hospitalization for supportive care: full bloodwork, 4DX/tick panel, UA, Urine culture, AUS in AM - owner declined due to finances

Owner will monitor at home and follow up with Community Animal Hospital tomorrow and receive an AUS there on Wednesday

Initial and current therapy: None

Diagnostics completed: None

Diagnostics pending: None

**CLE Case 4 biopsy report notes:**

Microscopic findings:

2 sections (more severely affected) have a remarkable lichenoid interface superficial dermatitis that somewhat extends into the follicular epithelium and adnexa, and obscures the dermoepidermal junction. Sebaceous glands when present appear spared. The inflammatory cell population is lymphoplasmacytic, histiocytic, and neutrophilic sometimes with the formation of intraepidermal foci. Occasional individual apoptotic keratinocytes are noted. In these sections, there is also focally extensive epidermal ulceration, hyperplasia and suppurative dermatitis. Pigmentary incontinence is also noted.

2 sections (less severely affected). The lichenoid pattern is less prominent as compared to the more severely affected sections above, but is still observed and lymphocytes are within the basal layer. There is also lymphoplasmacytic superficial dermatitis, edema, and focal superficial dermal fibrosis. Scattered non-degenerate neutrophils are noted in the dermis, and marginated in the blood vessels. In addition, in this specimen, the inflammation also extends into the adnexa and one follicular opening is particularly affected by suppurative inflammation.

Comment:

The biopsy features show a lichenoid-interface dermatitis affecting epidermal and follicular epithelium and there is some degeneration of the basal cell layer. Based on the history provided and the microscopic appearance, I speculate that the disease involves an immune-mediated process that appears directed towards the lower layers of the epithelium (this interpretation is based on the smaller nests of intraepidermal lymphocytes and the relative abundance of plasma cells within the superficial dermis). Interpretation is complicated by ulceration, suppurative inflammation and intralesional cocci, and it is difficult to ascertain what role the neutrophils have here.

Pigmentary incontinence is observed, which fits with the depigmentation noted clinically.

The following helpful differential diagnoses were listed:

Mucocutaneous pyoderma: Some features are consistent

Discoid lupus erythematosus: Some features are consistent

Pemphigus erythematosus: Some features are consistent, but I do not observe clefts or substantial basal cell apoptosis in these sections

Lymphoma: Does not fit the microscopy

Hepatocutaneous syndrome: Does not fit the microscopy
